# My personal mutanome: a computational genomic medicine platform for searching network perturbing alleles linking genotype to phenotype

**DOI:** 10.1186/s13059-021-02269-3

**Published:** 2021-01-29

**Authors:** Yadi Zhou, Junfei Zhao, Jiansong Fang, William Martin, Lang Li, Ruth Nussinov, Timothy A. Chan, Charis Eng, Feixiong Cheng

**Affiliations:** 1 Genomic Medicine Institute, Lerner Research Institute, Cleveland Clinic, Cleveland, OH 44195 USA; 2 Department of Systems Biology, Herbert Irving Comprehensive Center, Columbia University, New York, NY 10032 USA; 3 Department of Biomedical Informatics, Columbia University, New York, NY 10032 USA; 4 Department of Biomedical Informatics, College of Medicine, Ohio State University, Columbus, OH 43210 USA; 5 Computational Structural Biology Section, Basic Science Program, Frederick National Laboratory for Cancer Research, National Cancer Institute at Frederick, Frederick, MD 21702 USA; 6 Department of Human Molecular Genetics and Biochemistry, Sackler School of Medicine, Tel Aviv University, 69978 Tel Aviv, Israel; 7 Center for Immunotherapy and Precision Immuno-Oncology, Lerner Research Institute, Cleveland Clinic, Cleveland, OH 44195 USA; 8 Department of Molecular Medicine, Cleveland Clinic Lerner College of Medicine, Case Western Reserve University, Cleveland, OH 44195 USA; 9 Case Comprehensive Cancer Center, Case Western Reserve University School of Medicine, Cleveland, OH 44106 USA; 10 Taussig Cancer Institute, Cleveland Clinic, Cleveland, OH 44195 USA; 11 Department of Genetics and Genome Sciences, Case Western Reserve University School of Medicine, Cleveland, OH 44106 USA

**Keywords:** Mutanome, Protein-protein interaction, Edgetic, Nodetic, Somatic mutations, Precision cancer medicine

## Abstract

Massive genome sequencing data have inspired new challenges in personalized treatments and facilitated oncological drug discovery. We present a comprehensive database, My Personal Mutanome (MPM), for accelerating the development of precision cancer medicine protocols. MPM contains 490,245 mutations from over 10,800 tumor exomes across 33 cancer types in The Cancer Genome Atlas mapped to 94,563 structure-resolved/predicted protein-protein interaction interfaces (“edgetic”) and 311,022 functional sites (“nodetic”), including ligand-protein binding sites and 8 types of protein posttranslational modifications. In total, 8884 survival results and 1,271,132 drug responses are obtained for these mapped interactions. MPM is available at https://mutanome.lerner.ccf.org.

**Supplementary Information:**

The online version contains supplementary material available at 10.1186/s13059-021-02269-3.

## Background

Recent advances in high-throughput sequencing have led to the availability of hundreds of thousands of exomes and genomes, which contain billions of single-nucleotide variants including millions of missense variants [1, 2]. The Cancer Genome Atlas (TCGA, https://www.cancer.gov/tcga) program has characterized the genomes/exomes of > 11,000 patients across 33 cancer types. The Catalogue of Somatic Mutations in Cancer (COSMIC) is a major somatic mutation database in cancer [3]. cBioPortal allows users to visualize, analyze, and download large-scale cancer genomic data sets [4, 5]. Even though these data and web resources have greatly facilitated cancer research and drug discovery, better interpretation of the pathogenicity of variants critical for the advancement of precision medicine is under-studying, marring the understanding of the consequences of genetic variants in clinical settings [6]. Typical computational approaches can only identify a small portion of the pathogenic variants with the high confidence that is required for clinical decision making. Identification and prioritization of causal and clinically actionable mutations is essential to understand their roles in tumorigenesis and disease progression, discover new biomarkers, and offer biologically relevant drug targets [7].

**Fig. 1:**
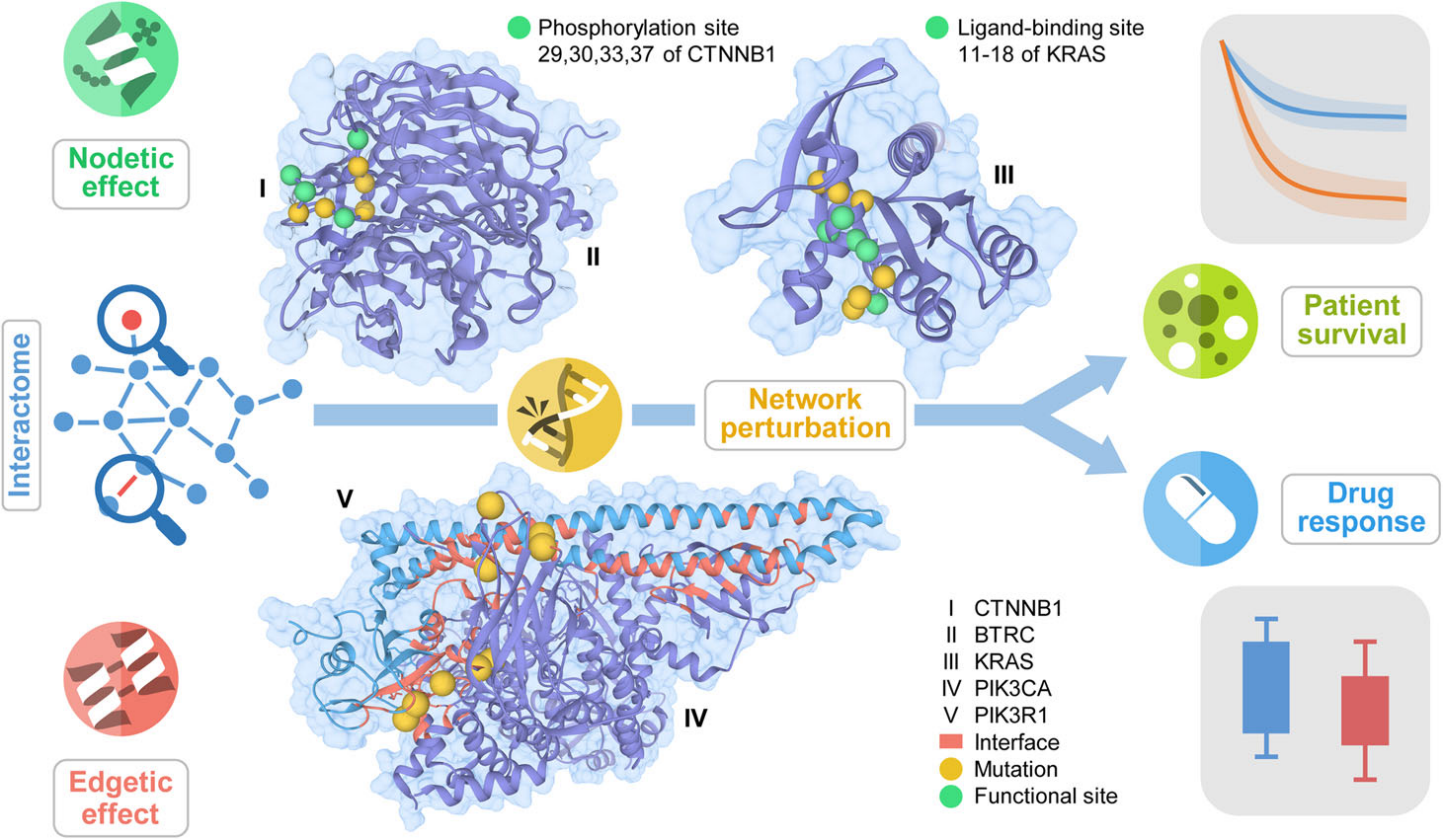
The overall design of My Personal Mutanome. The human interactome is a network of protein-protein interactions where proteins are the nodes and interactions are the edges. Perturbations, such as those originated from mutations, alter the networks by either directly affecting the nodes (nodetic) or affecting the edges (edgetic). Nodetic mutations are those that can affect the function of a protein, such as post-translational modification and ligand-binding. For example, the phosphorylation sites of catenin beta-1 (encoded by *CTNNB1*) (I) are affected by the mutations at the same sites or in close vicinity. The ligand-binding site of KRAS (III) can also be affected by the nodetic mutations. Edgetic mutations are those on the PPI interfaces that perturb the interaction. For example, several mutations on the PPI interface (red) of PIK3CA (IV) and PIK3R1 (V) perturb the interaction. Nodetic and edgetic network perturbations by mutations may promote tumorigenesis, results in altered patient survival and drug responses. By integrating the survival and drug responses and 3D structural information, MPM helps to identify actionable mutations and provides implication of the mechanisms at the human interactome level

Genes and proteins do not function independently, but in complex, interconnected networks and pathways [8–10]. The human interactome is a network of proteins (nodes) connected by their physical interactions (edges) (Fig. 1 and Additional file 1: Fig. S1). Mutations perturb the network either by directly altering the normal functions of the proteins (“nodetic” effect), such as via post-translational modification and ligand-binding, or by altering the protein-protein interactions (PPIs) (“edgetic” effect). Theoretically, in the human interactome, nodetic effect refers to the effect that a mutation directly knockout or knockdown a gene/protein function and consequently removing the protein and all its edges [8, 9]; alternatively, mutation effects can also be PPI specific, causing removal or gain of specific PPIs, known as edgetic effect [8–10]. Nodetic and edgetic network perturbations by mutations have been shown to promote tumorigenesis and disease progression [11] and result in altered patient survival and drug responses. Our previous studies have shown that in cancer, somatic missense mutations tend to be enriched at protein functional sites such as protein-ligand binding sites [12], protein allosteric sites [13], and phosphorylation sites [14]. Investigation of the nodetic effects of mutations could help uncover likely driver mutations with mechanistic implications and offer personalized drug treatments [6]. Studies have shown that disease-related mutations tend to localize in PPI interfaces and perturb the interactions of the same mutated protein with multiple partners [10, 15]. Recent ongoing community efforts have completed the mapping of the human interactome and provided the increasing availability of structural genomic information on PPIs from diverse sources including the PDB [16], Interactome INSIDER [17], and Interactome3D [18]. These protein structural genomic resources offer unexpected opportunities for accelerating interpretation of biological and functional consequences of cancer mutations for precision cancer medicine from systems biology perspectives [6, 19]. In our recent study, we found that somatic missense mutations were highly enriched in PPI interfaces compared to non-interfaces via analysis of over 10,000 whole exomes across 33 cancer types [20]. We further showed that PPI interface mutation analysis provided likely causal relationships in tumorigenesis and experimentally validated functional effects of PPI interface mutation using a systematic binary interaction assay [8–10] and cell line-based functional assays [20]. In summary, all previous observations from our groups and other studies provide functional proof-of-concept of both nodetic and edgetic effects of somatic mutations in human cancer. These results motivate us to develop a systems biology tool for querying such nodetic and edgetic mutations in the human interactome, which will be valuable for identifying novel functional mutations/genes, drug targets, and pharmacogenomics biomarkers for precision cancer medicine.

**Fig. 2:**
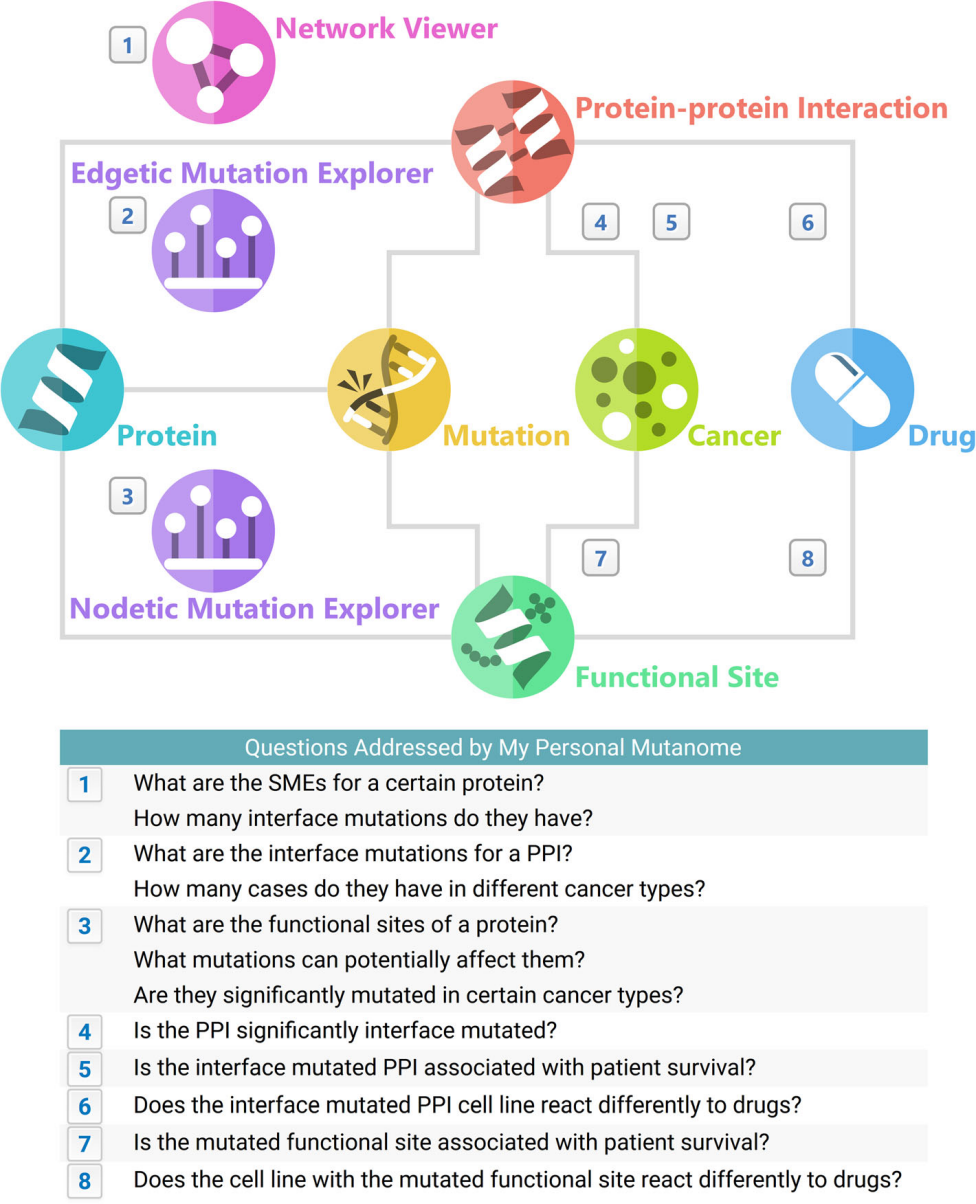
Information architecture and questions addressed by My Personal Mutanome. This figure illustrates the several main entities (such as protein and drug) and their relationships (indicated by edges) in My Personal Mutanome. Users can browse through all the information by following the architecture shown here. Three visualization tools were developed: “Network Viewer,” which shows the subnetwork of the significantly mutated edges of a certain protein; “Nodetic Mutation Explorer,” which bridges the mutations to the functional sites; and “Edgetic Mutation Explorer,” which displays the mutations on the protein-protein interaction interfaces. Both “Nodetic” and “Edgetic” come with a built-in protein 3D structure viewer. Drug responses and cancer patient survival were computed for the nodetic and edgetic mutations. Numbers indicate the questions addressed by the information and visualization provided by My Personal Mutanome

We therefore developed My Personal Mutanome (MPM), a comprehensive database of nodetic and edgetic effects of somatic mutations across 33 cancer types/subtypes. Figure 2 summarizes the main data entities and their relationships, as well as eight main questions addressed by the data and tools provided by MPM. We integrated 490,245 somatic mutations, 121,172 physical PPIs, and 535,182 functional sites composed of 8 varieties: acetylation (43,764), malonylation (4476), methylation (14,649), O-linked glycosylation (4228), phosphorylation (276,738), succinylation (1665), ubiquitination (100,246), and ligand binding (89,416). We systematically mapped all the mutations to 94,563 PPIs and 311,022 functional sites. For the human interactome, we combined data from three sources to build a comprehensive PPI interface database. Using statistical methods, we systematically identified putative SMEs (Significantly Mutated Edges, also termed oncoPPIs) which harbor a statistically significant excess number of somatic missense mutations at PPI interfaces (see Methods). We then performed survival and drug response analysis for these mappings. MPM offers three interactive visualization tools that provide 3D views of somatic mutations in the context of the human interactome network (nodetic and edgetic) with their clinical (survival) and drug responses. MPM is expected to facilitate the identification of actionable mutations for tumorigenesis and personalized treatments at the human interactome level. Collectively, it offers network-based diagnosis and pharmacogenomics approaches to understand complex genotype-phenotype relationships and therapeutic responses in the clinical settings. MPM is available at https://mutanome.lerner.ccf.org.

## Construction and content

### Data collection

#### Genes and proteins

Gene information was retrieved from HGNC (https://www.genenames.org/) [21]. Protein information was downloaded from UniProt (https://www.uniprot.org/uniprot/) [22]. Gene and protein mapping were downloaded using the “Retrieve/ID mapping” tool from UniProt. A total of 21,759 proteins with somatic mutations from TCGA database were mapped to 19,149 protein coding genes from HGNC.

#### Human protein-protein interactome

We used the human protein-protein interactome from our previous studies [20, 23]. Briefly, high-quality PPIs were assembled from 15 commonly used databases that include five types of evidence: yeast-two-hybrid system, protein 3D structures, literature-derived kinase-substrate interactions, literature-derived signaling networks, and affinity-purification mass spectrometry.

#### PPI interfaces

PPI interfaces were combined using three sources: PDB (http://www.rcsb.org/) [16], ECLAIR (http://interactomeinsider.yulab.org/) [17], and Interactome3D (https://interactome3d.irbbarcelona.org/) [18]. PDB provides many resolved 3D structures that contains both interacting proteins in some PPIs. ECLAIR and Interactome3D utilizes machine learning-based approaches and homology modeling to predict PPI.

#### Post-translational modification sites

Seven types of post-translational modifications sites (acetylation, malonylation, methylation, O-linked glycosylation, phosphorylation, succinylation, ubiquitination) were downloaded from four databases: dbPTM (http://dbptm.mbc.nctu.edu.tw/) [24], PhosphoSitePlus (https://www.phosphosite.org/homeAction.action) [25], Phospho.ELM (http://phospho.elm.eu.org/) [26], and PTMD (http://ptmd.biocuckoo.org/) [27].

#### Ligand-binding site

We downloaded the ligand-binding site data from BioLiP (https://zhanglab.ccmb.med.umich.edu/BioLiP/) [28], which offers high-quality manually curated ligand-protein binding information.

#### Somatic mutations and cancer patient information

We downloaded 10,861 human exomes (the tumor-normal pairwise somatic mutation data) across 33 cancer subtypes/types and their survival information from TCGA GDC Data Portal (https://portal.gdc.cancer.gov/). We integrated the results of four scoring methods for the evaluation of pathogenic impacts of the mutations. The sorting intolerant from tolerant (SIFT) and polymorphism phenotyping v2 (PolyPhen-2) were computed using ANNOVAR [29]. Combined Annotation Dependent Depletion (CADD) scores were downloaded from https://cadd.gs.washington.edu/ [30]. FoldX scores (change in structure stability between mutated and reference structure, ddG) were downloaded from http://www.mutfunc.com/ [31].

#### Drug responses

A total of 251 drugs tested in 1074 cancer cell lines with half maximal inhibitory concentration (IC_50_) data points were downloaded from GDSC (http://www.cancerrxgene.org/) [32]. For each drug, we constructed a drug-response vector consisting of *n* IC_50_ values from treatment of *n* cell lines. Then, drug-response vector was modeled as a linear combination of the tissue of origin of the cell lines, screening medium, growth properties, and the status of a genomic feature. A genomic feature-drug pair was tested only if the final drug-response vector contained at least three positive cell lines and at least three negative cell lines. Effect size was quantified through the Cohen’s *d* using the difference between two means divided by a pooled standard deviation for the data. The resulting *p* values were corrected by Benjamini-Hochberg method.

### Nodetic and edgetic effects evaluation

We first mapped all somatic mutations to the PPI interfaces and protein functional sites. Using the interface information, all mutations on a certain PPI were classified as either interface mutations or non-interface mutations. For functional sites, a 15- or 7-amino acid window was applied for each site (from position − 7 to + 7 centered at the post-translational modification sites, and -3 to +3 at the ligand-binding sites) to screen for mutations as described in previous studies [12, 14].

For each type of functional site (e.g., phosphorylation site), we tested whether the mutations of gene *g*_*i*_ in a certain cancer type are significantly enriched near the functional site. We computed the *p* value using binomial distribution:

1 $$ P\left(X\ge k\right)=1-P\left(X<k\right)=1-\sum \limits_{x=0}^{k-1}\left(\frac{T}{x}\right){p}_{g_i}^x{\left(1-{p}_{g_i}\right)}^{T-x} $$

where *T* is the total number of mutations observed in the protein product of gene *g*_*i*_, and ${p}_{g_i}$ is the estimated mutation rate for the window flanking this functional site under the null hypothesis. Using W to represent the window size and ${L}_{g_i}$ the length of protein product of gene *g*_*i*_, ${p}_{g_i}$ was calculated as

2 $$ {p}_{g_i}=\frac{W}{L_{g_i}} $$

Next, we computationally identified putative SMEs harboring a statistically significant excess number of missense mutations at PPI interfaces in pan-cancer analysis and individual cancer analysis under the null hypothesis that the mutations were randomly distributed on the sequences of two proteins of gene *g*_1_ and *g*_2_ in each PPI. Similarly, using ${L}_{g_i}$ to represent the length of protein product of gene *g*_*i*_, we calculated the mutation rate in the interface of *g*_*i*_ as

3 $$ {p}_{g_i}=\frac{L_{g_i\ast }}{L_{g_i}} $$

where ${L}_{g_i\ast }$ is the interface length. After using binominal test to assess the significance of enrichment of mutations in the interfaces of *g*_1_ and *g*_2_, we used the product of two *p* values *P*_1_ and *P*_2_ to represent the significance of mutation enrichment in this PPI interface.

### Survival analysis

Kaplan-Meier survival analysis (adjusting age, tumor stages, and other confounding factors) was performed with the patient survival data from TCGA using the R (3.6.3) (https://www.r-project.org/).

### Website implementation

MPM was implemented with Python (3.7.2) (https://www.python.org/) framework Django (2.2.2) (https://www.djangoproject.com/) on the server backend. Django adopts a Model-Template-View pattern that decouples the database, content, and website logic, which allows rapid implementation of website features and provides high reusability of each component. SQLite (https://www.sqlite.org/) was used for the relational database. We decided to implement the views such that they respond to user requests with pure JSON format data. This architecture enables users to access all our data through user programs so that MPM can be integrated in their pipelines. HTML, CSS, and JavaScript were used for the frontend. The frontend was heavily programmed in JavaScript to offer the user a smooth experience with highly interactive visualization tools. AJAX was used to asynchronously retrieve data in JSON format and populates the web page on user requests. Network visualization was implemented using Cytoscape.js [33]. The PDB viewer was implemented based on PV [34]. Nodetic and edgetic mutation explorers were implemented with HTML canvas and JavaScript. MPM is hosted by the Cleveland Clinic Lerner Research Institute Computing Services.

## Utility and discussion

### Database overview

We have assembled and processed all the data, including 21,759 proteins, 490,245 somatic mutations, and 544,692 mutation cases (count excludes those neither mapped to PPIs nor functional sites), 121,172 PPIs, drug responses of 251 drugs tested in 1074 cancer cell lines, 41,843 PDBs, and 535,182 protein functional sites for protein-ligand binding and across 7 types of protein post-translational modifications (PTMs): acetylation, malonylation, methylation, o-linked glycosylation, phosphorylation, succinylation, ubiquitination (Table 1).

**Table 1:** My Personal Mutanome database statistics

**Entities**		
Protein	21,759	
Mutation	490,245	
	Interface	72,953
	Non-interface	417,292
Mutation case	544,692	
	Interface	85,484
	Non-interface	459,208
PPI	121,172 from 3 sources:	
	PDB	4112
	Interactome3D	2891
	ECLAIR	114,169
Functional site	535,182 in 8 types:	
	Acetylation	43,764
	Malonylation	4476
	Methylation	14,649
	O-linked glycosylation	4228
	Phosphorylation	276,738
	Succinylation	1665
	Ubiquitination	100,246
	Ligand binding	89,416
TCGA	11,315 cases in 33 cancer types	
Drug	251	
PDB	41,843	
	95,599 PDB chains for 6583 proteins	
	20,132 PDBs for 9589 PPI	
	5126 PDBs for 6482 PPI (hetero)	
	17,595 PDBs for 3107 PPI (homo)	
**Relations**		
	PPI (edgetic)	Protein functional site (nodetic)
Mutation	775,524 mapped interface mutations	450,685 mapped mutations
Cancer	7285 survival analyses 2,087,382 oncoPPI tests	1599 survival analyses
Drug	252,275 drug responses	1,018,857 drug responses

*PPI* protein-protein interaction, *Hetero PPI* PPI consisting of different proteins, *Homo PPI* PPI of the same protein

We mapped all the mutations to the PPIs and protein functional sites. We found that somatic missense mutations are significantly enriched in ligand-binding sites (Additional file 1: Fig. S2) and phosphorylation sites (Additional file 1: Fig. S3) compared to non-ligand-binding sites and non-phosphorylation sites, respectively, across all 33 cancer types/subtypes, which are consistent with our previous findings [12–14]. Survival analyses were performed by dividing patients into a wild-type group (not interface mutated for edgetic, or not mutated on the functional sites for the nodetic) and a mutant group. Drug response comparisons were conducted in the same manner (see Methods). For nodetic effects, we calculated the impact of mutations at the functional site type level to provide the user an overview of which type of functional site is significantly affected by somatic missense mutations in a specific cancer type. For edgetic effects, we performed oncoPPI test for all the PPIs in pan-cancer and 33 individual cancer types/subtypes, which will enable the user to quickly search potentially mutation-perturbing PPIs for a specific cancer type or pan-cancer from the human interactome.

PDBs were mapped to proteins at the residue level, enabling a simple and quick structural examination for each resolved residue. In addition, 5126 and 17,595 PDBs were mapped to 6482 heterodimers (hetero PPIs, two different proteins) and 3107 homodimers (homo PPIs, interaction of two identical proteins), respectively. When available, PDBs containing both proteins in a PPI are highlighted and prioritized for display, which helps to visualize the PPI interfaces to illustrate likely functional (hotspot or weak driver) mutations and their potential structural effects.

All data and results have been integrated into a relational database. Our website utilizes the relations between several main entities (protein, mutation, PPI, functional site, and drug) to navigate the users to the information they are searching for, which is explained in the next section.

### Web interface

**Fig. 3:**
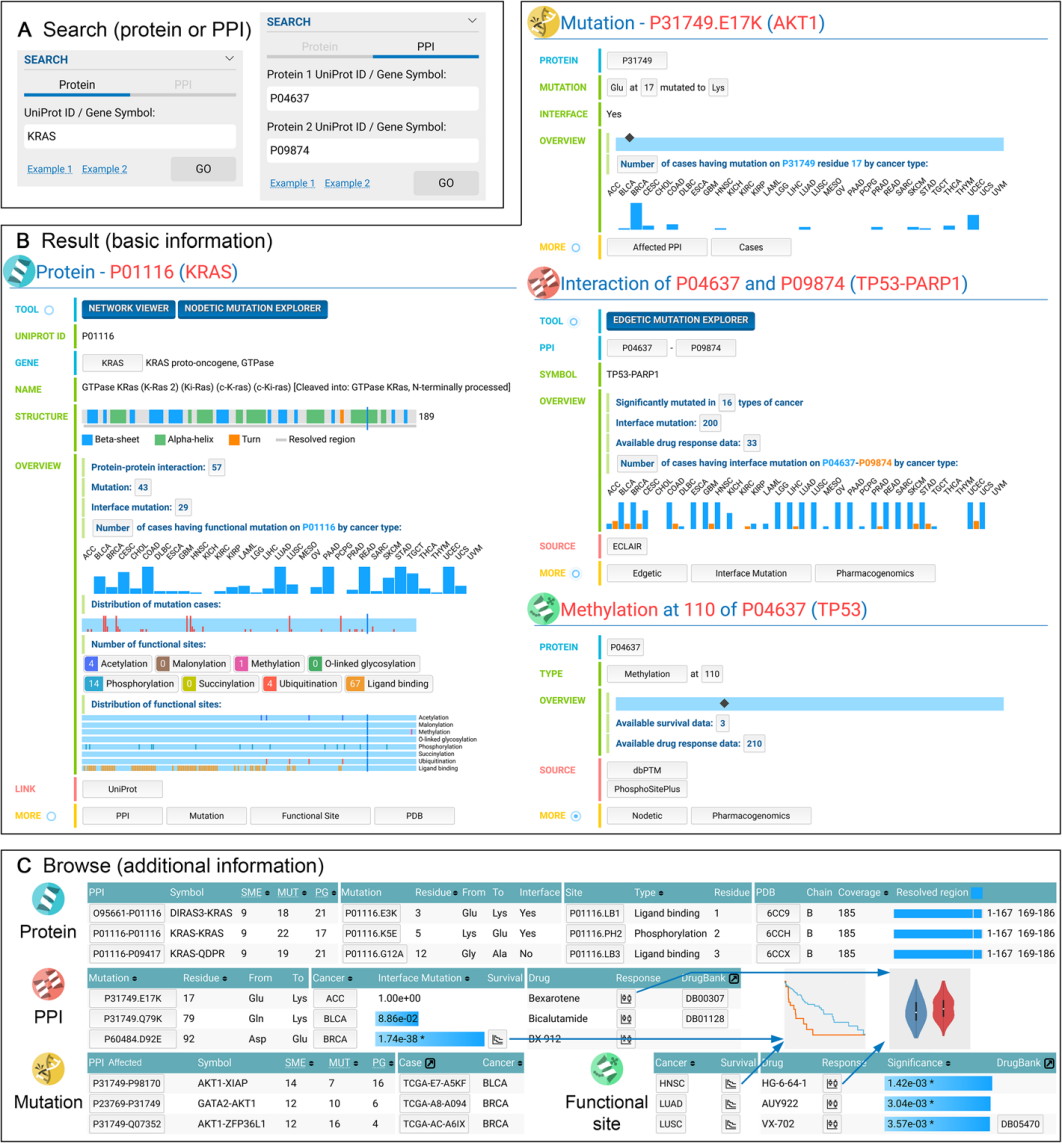
Overview of the web interface. My Personal Mutanome has an all-in-one interface that allows users to search (**a**), view results (**b**, **c**), and visualize nodetic/edgetic mutations (Figs. 4 and 5) in the same web page, improving the smoothness in navigating through the database and removing the needs for switching between browser tabs. Users can use both UniProt ID and gene symbol to search for a protein or a specific PPI (**a**), which loads the basic information page (**b**). These pages provide an overview of the data that are available for the proteins, PPIs, mutations, and functional site in My Personal Mutanome. (**c**) Additional information such as all the mapped mutations for a specific PPI can be loaded on user request. Survival and drug responses are found by loading the “Edgetic” and the “Pharmacogenomics” information on the PPI and functional site pages. By clicking the buttons, users can browse through all the related information progressively

The main web interface, Mutanome Explorer, is where users will perform data exploration and visualization in MPM. Mutanome Explorer is an all-in-one interface that utilizes the highly relational nature of the data. This permits smooth navigation of the data with minimal typing and searching. All data types are loaded onto the same web page, organized by tabs. Upon entering Mutanome Explorer, users will see an embedded help page with detailed instructions. The entry points to the database are protein and PPI searches (Fig. 3a), which accepts both UniProt ID and gene symbol. Then, a protein/PPI page is loaded. Protein, mutation, PPI, and functional site are the four major entities, with each having its own page (Fig. 3b). On each page type, several buttons at the bottom (in the “More” section) list the relevant related entities (Fig. 3c). For example, clicking “Mutation” on a protein page lists the mutations, and clicking a mutation in the list loads a new page for the mutation. Clicking the “Edgetic” and “Nodetic” shows the survival results for the PPI and functional site, respectively. The “Pharmacogenomics” button lists the drug response comparisons. Figure 3c shows an example for each type of list.

**Fig. 4:**
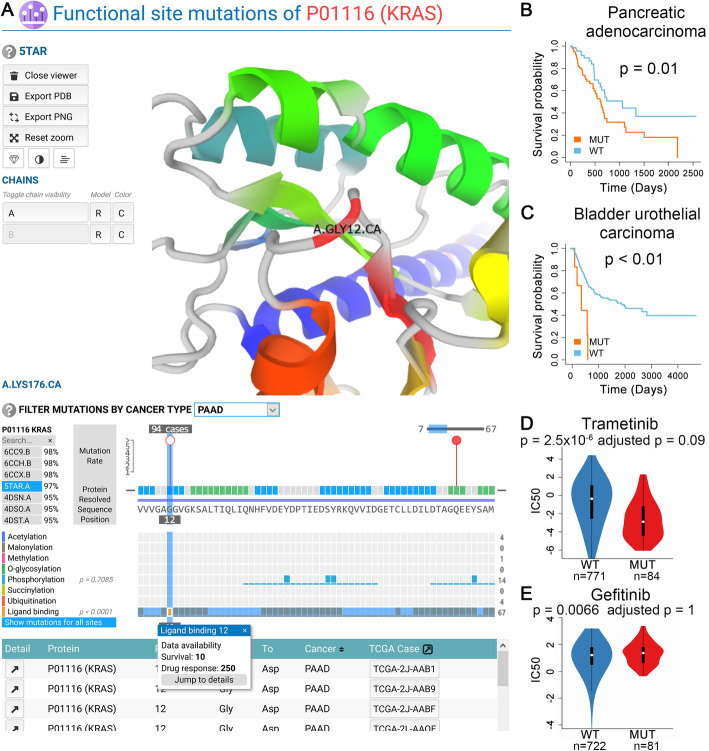
A nodetic use case. The usage of “Nodetic Mutation Explorer” is demonstrated using the Gly12 mutation of KRAS that may affect the ligand-binding (**a**) and clinical responses (**b**–**e**). **a** “Nodetic Mutation Explorer” has four components. From top to bottom, they are (i) a 3D structure viewer for PDBs, (ii) protein 2D viewer with mutations that is filterable by cancer types and functional site types, (iii) functional site visualization that is synchronized with (ii), and (iv) a table of cases for a specific mutation. Upon clicking a mutation, the corresponding residue is automatically centered in the PDB viewer, and all the cases are listed in the table. Once a mutation and functional site is identified, users will find the survival and drug responses information on the information page of the functional site. In this case, patient survival of pancreatic cancer (**b**) and bladder urothelial cancer (**c**) are significantly associated with Gly12 mutations that potentially alter the ligand-binding of KRAS. Additionally, drug responses are available for this site, and examples are shown in **d** and **e**

**Fig. 5:**
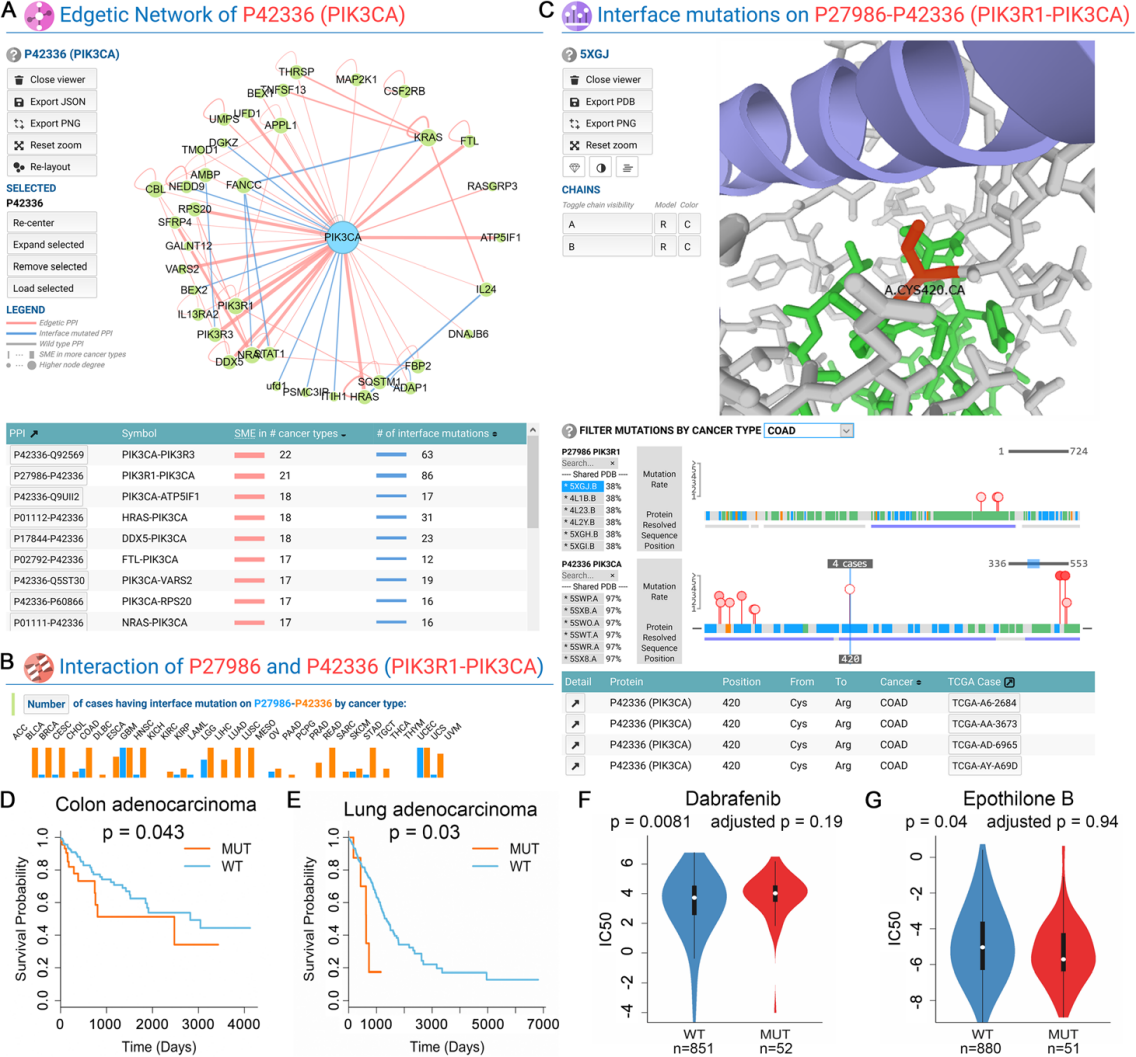
An edgetic use case. “Network Viewer” can be used to identify potential driver edges in cancer (**a**). A subnetwork is shown for a specific protein, which contains all the known PPIs among the protein and its neighbors. oncoPPIs are in red and those are not oncoPPIs but have interface mutations are in blue. Clicking a PPI will load the information page of that PPI. The mutation distribution in different cancer types of a PPI page is shown in **b**. The “Edgetic Mutation Explorer” (**c**) has a PDB viewer, a sequence/mutation viewer for each protein in the PPI, and a table that lists the mutations. The interaction of PIK3R1-PIK3CA is used as an example. Both proteins are frequently mutated in multiple cancer types (**b**). Recurrent interface mutations (Cys420Arg of PIK3CA) are revealed (**c**) with significant clinical responses (**d**–**g**)

Several interactive visualization tools were implemented to facilitate the discovery of actionable mutations. “Network Viewer” helps to identify significantly mutated PPIs from the human interactome. “Nodetic Mutation Explorer” shows the mutations in a selected cancer type with 3D structure that potentially affects the functional sites (Fig. 4). “Edgetic Mutation Explorer” displays the interface mutations on a PPI with 3D structure and the mutation cases in various cancer types (Fig. 5). Usage of these tools is explained with two case studies next.

### Use case—nodetic example

KRAS provides an example demonstrating how to use the “Nodetic Mutation Explorer” to identify clinically relevant and actionable functional site mutations. The *KRAS* gene encodes a small ATPase that acts as a binary switch that controls signal transduction in cells [35]. It is one of the most mutated oncogenes in multiple cancer types [36–38].

We first searched for “KRAS” (or UniProt ID “P01116”) in the protein search box to load the protein information page. In the “OVERVIEW” section, KRAS was most highly mutated in pancreatic adenocarcinoma (PAAD), colon adenocarcinoma (COAD), and rectum adenocarcinoma (READ). Out of the 185 PAAD TCGA cases, 101 cases (55%) had mutations on KRAS that mapped to PPI interfaces or functional sites. For COAD and READ, the numbers are 163 out of 461 (35%) and 49 out of 172 cases (28%), respectively. Next, clicking on “NODETIC MUTATION EXPLORER,” we entered the visualization tool. There are four sections in the tool (Fig. 4a). From top to bottom, they are (i) PDB viewer; (ii) protein sequence and mutation distribution viewer; (iii) functional site viewer organized by types, aligned with the protein sequence in (ii); and (iv) a table that shows the cases when a mutation is selected. The PDB file that has the highest coverage for KRAS was loaded automatically. By default, mutations in all cancer types are shown, but they can be filtered by a specific cancer type. We immediately noticed that Gly12 (439 cases), Gly13 (89 cases), Gln61 (50 cases), and Ala146 (29 cases) were the most mutated residues in pan-cancer. These mutations are established as oncogenic [39–41].

KRAS is strongly associated with pancreatic tumorigenesis [42, 43]. Having the highest mutation rate of KRAS compared to other cancer types, PAAD was selected to search the mutations. Ligand-binding sites were significantly affected by mutations (Fig. 4a, orange row, *p* < 0.0001). Gly12 and Gln61 had 94 and 7 mutations cases, respectively, in PAAD. These mutation sites are also ligand-binding sites. Clicking the orange rectangle for ligand-binding site 12 opens the information page of this site. Examining the survival analysis results, the Gly12 mutations of ligand-binding site 12 is significantly associated with patient survival in PAAD (Fig. 4b, *p* = 0.01). These mutations were also associated with bladder urothelial carcinoma (BLCA) patient survival (Fig. 4c, *p* < 0.01). Next, by examining the drug response results, we found that mutations on ligand-binding site 12 altered several drugs’ responses. Trametinib is a MEK inhibitor that has been approved by FDA for cancer treatment [44]. Trametinib had a lower IC_50_ in mutant cell lines (Fig. 4d, *p* = 2.5 × 10^−6^), showing that Gly12 mutant cell lines were more sensitive to trametinib. This is consistent with the findings in a previous study [39]. In addition, there have been a number of clinical trials involving trametinib alone [45] or in combination with other drugs [46] for non-small cell lung cancer. Another drug, gefitinib, targets the epidermal growth factor receptor (EGFR) by inhibiting the tyrosine kinases associated with EGFR [47]. It has been approved for non-small cell lung cancer treatment. KRAS mutations are associated with a lack of sensitivity to gefitinib [48]. Gefitinib showed a higher IC_50_ in mutant cell lines (Fig. 4e, *p* = 0.0066), confirming that KRAS mutants are more resistant to gefitinib.

### Use case—edgetic (oncoPPIs) example

The interaction between the two phosphatidylinositol 3-kinase (PI3K) subunits provides an example of a mutation edgetic effect and the “Network Viewer” and “Edgetic Mutation Explorer.” PI3K pathway, which regulates multiple cellular events such as cell growth, apoptosis, and survival [49], is frequently dysregulated in cancer. *PIK3CA* is also one of the most commonly mutated genes [50]. The PI3Kα isoform is composed of two subunits: the catalytic subunit p110α encoded by the *PIK3CA* gene and the regulatory subunit p85 encoded by the *PIK3R1* gene.

In the PIK3CA page, PIK3CA is highly mutated (153 mutations and 1278 mutant cases), in multiple cancer types. There are 39 PPIs for PIK3CA, among which, 31 PPIs are potential oncoPPIs in at least one cancer type. We then click the “NETWORK VIEWER” button (Fig. 5a) to visualize these PPIs. PIK3CA-PIK3R3 is an oncoPPI in the highest number of cancer types (22 types). However, it lacked a PDB structure containing both interacting proteins to show the PPI interface mutations. PIK3CA-PIK3R1 are another potential oncoPPIs in 21 cancer types (second highest) having 86 interface mutations.

After clicking on the “P27986-P42336” button, MPM will load the information for PIK3CA-PIK3R1. Although both proteins were mutated in various cancer types, most of the interface mutations were detected on PIK3CA (Fig. 5b, orange bars). By exploring the “More” section, we also found that interface mutations of PIK3CA-PIK3R1 were associated with patient survival of COAD (Fig. 5d, *p* = 0.043) and lung adenocarcinoma (LUAD) (Fig. 5e, *p* = 0.03). Several drug responses are affected by the interface mutations. Dabrafenib is a competitive kinase inhibitor of BRAF for the treatment of melanoma [51]. It has been used in combination with trametinib for the treatment of non-small-cell lung cancer [52] and anaplastic thyroid cancer [53]. Dabrafenib had a higher IC_50_ in mutant tumor cell lines (Fig. 5f, *p* = 0.0081) compared to wild-type cell lines, consistent with a recent study that mutant PIK3CA and AKT3 increases the resistance of melanoma cells to BRAF inhibitor dabrafenib [54]. Epothilone B is a microtubule inhibitor used for the treatment of multiple myeloma [55]. It shows a lowered IC_50_ in mutant tumor cell lines (Fig. 5g, *p* = 0.04). A previous study reported that epothilone B enhanced the apoptotic effects of ABT-737 through the PI3K/AKT/mTOR pathway [56]. Altogether, these findings could be used to guide the treatment for patients with interface mutations on PIK3CA-PIK3R1.

Next, we examined PIK3CA-PIK3R1 interface mutations that may be responsible for the COAD patients’ survival using “Edgetic Mutation Explorer” (Fig. 5c). PDB accession 5XGJ was automatically selected, which contained both PIK3CA (chain A) and PIK3R1 (chain B). 5XGJ covered all the interface mutations, as indicated by the purple bars. For pan-cancer, several mutation hot-spots were revealed on PIK3CA: Glu545 (316 cases), Glu542 (183 cases), Gln546 (54 cases), Asn345 (48 cases), Cys420 (26 cases), and Glu453 (25 cases). Gly376 (13 cases) and Asn564 (10 cases) were the most frequently mutated spots. Some of these were reported previously in several cancer types [57–62]. We then set the cancer type to COAD. Glu545 (38 cases) and Glu542 (14 cases) of PIK3CA were the most populated mutation sites in COAD patients. Cys420 was highly mutated in pan-cancer (26 cases), and had 4 cases in COAD which were all mutated to arginine. By clicking Cys420, we also found that Cys420 on PIK3CA was directly pointing towards PIK3R1, with a distance less than 3.5 angstrom (Å) to the nearest residues on PIK3R1.

## Limitation and future directions

We acknowledge several limitations. We assembled PPI interface data from known protein complex structures, homology models, and machine learning-based computational computation as the crystal structure-derived data is very limited. Although we showed that somatic missense mutations were significantly enriched in computationally predicted PPI interfaces [20], further improving the quality of PPI interfaces (including cryogenic electron microscopy (cryo-EM) structure) are highly needed in the future. The computation for SMEs did not take the sequence composition and amino acid specific mutation rate into consideration. However, when we recomputed the significance of the SMEs, we found that the new results are highly consistent to the original results, suggesting a small effect by accounting for these factors (Additional file 1: Fig. S4). Third, we applied a 15-amino acid window (± 7) to screen for mutations for phosphorylation sites. Mutations that do not directly overlap with the functional sites may not have nodetic or edgetic effect. However, if we only consider the exact position (i.e., ± 0), the analysis will be underpower due to the sparsity of data. In addition, we evaluated different window sizes (± 0, ± 1, ± 3, and ± 5) for phosphorylation sites and found that ± 3, ± 5, and ± 7 produced similar results (Additional file 1: Fig. S5). It is unclear whether the presence of the mutation may have a functional effect on phosphorylation-based singling networks. We tested how phosphorylation site mutations have functional impact on signal networks using proteogenomics data from The National Cancer Institute’s Clinical Proteomic Tumor Analysis Consortium (CPTAC) using COAD as an example. We found that protein quantification is significantly lowered in mutated phosphorylation sites than wild type sites in COAD (*p* = 0.012, Additional file 1: Fig. S6); yet, protein quantification is relatively lowered in mutated phosphorylation sites than wild type sites in breast (BRCA, *p* = 0.052) and ovarian cancer (OV, *p* = 0.18). The differences of protein quantification in wild type and mutated phosphorylation sites in BRCA and OV are not significant, which could be due to an insufficient number of tumor samples and overall low mutation load in BRCA and OV. These observations reveal potential functional impacts of phosphorylation site mutations; further experimental validation is highly warranted using large-scale proteogenomics and phosphoproteomics datasets from cancer cell lines or tumor tissues. Finally, the human interactome is still incomplete and PPIs may have literature bias. We will continue updating the human interactome into the database, especially including more unbiased systematic PPIs data [20]. In addition, we will offer functions for selecting smaller window sizes for the functional sites. Future updates for MPM will be focused on providing more complete, high-quality human interactome (including protein-DNA/RNA interactions as well), functional sites, and proteogenomics data from CPTAC. We will integrate more human genome sequencing data, including Trans-Omics for Precision Medicine (TOPMed) Program [63], Alzheimer’s Disease Sequencing Project (ADSP) [64], and International Cancer Genome Consortium (ICGC) [65], to improve utilities of MPM by adding more personalized genome analyses.

## Conclusions

In summary, My Personal Mutanome offers a comprehensive database and powerful visualization tools that bridge the translational gap between large-scale genomic medicine studies and clinical outcomes. MPM offers rapid searching of actionable mutations and targets to guide personalized treatments and precision medicine drug discovery. By mapping mutations to PPI interfaces and protein functional sites and integrating clinical responses in terms of patient survival and drug response, MPM helps users identify cancer-driving and actionable missense somatic mutations associated with nodetic or edgetic effects in the scope of human protein-protein interactome and provides mechanistic and potential drug treatment implications. MPM will be updated annually to continue to provide the most complete data available.

## Supplementary Information

**Additional file 1: Supplementary figure S1-S6. Figure S1.** Diagram illustrating different types of nodetic versus edgetic perturbation leading to distinct phenotypes. **Figure S2**. Mutation rate is enriched in the ligand binding sites for these cancer types. **Figure S3**. Mutation rate is enriched in the phosphorylation sites for these cancer types. **Figure S4**. Correlation between the original results and new results by considering the amino acid specific background mutation rate and the amino acid composition for ligand-binding (LB) sites and post-translational modification (PTM) sites across 33 cancer types. **Figure S5**. Comparison of the results using different window sizes for the phosphorylation sites across all cancer types. **Figure S6**. Protein quantification is lowered in mutated phosphorylation sites than wild type sites in (A) breast invasive carcinoma, (B) colon adenocarcinoma, and (C) ovarian serous cystadenocarcinoma.

**Additional file 2.** Review history.

## Data Availability

My Personal Mutanome is available at https://mutanome.lerner.ccf.org to all users without any login or registration restrictions. The code for all mutation mapping and analysis can be found in https://github.com/ChengF-Lab/mutanome under the MIT License [66] and on Zenodo [67]. Gene and protein information was retrieved from HGNC (https://www.genenames.org/) [21] and UniProt (https://www.uniprot.org/uniprot/) [22]. PPI interface information was combined from three sources: PDB (http://www.rcsb.org/) [16], ECLAIR (http://interactomeinsider.yulab.org/) [17], and Interactome3D (https://interactome3d.irbbarcelona.org/) [18]. Protein functional sites were downloaded from dbPTM (http://dbptm.mbc.nctu.edu.tw/) [24], PhosphoSitePlus (https://www.phosphosite.org/homeAction.action) [25], Phospho.ELM (http://phospho.elm.eu.org/) [26], PTMD (http://ptmd.biocuckoo.org/) [27], and BioLiP (https://zhanglab.ccmb.med.umich.edu/BioLiP/) [28]. Somatic mutation and cancer patient information was downloaded from TCGA GDC Data Portal (https://portal.gdc.cancer.gov/). Mutation scores were retrieved from https://cadd.gs.washington.edu/ [30] and http://www.mutfunc.com/ [31]. Drug response data were retrieved from GDSC (http://www.cancerrxgene.org/) [32].
