# Supplementary material for: My personal mutanome: a computational genomic medicine platform for searching network perturbing alleles linking genotype to phenotype
Source: Genome Biol. 2021 Jan 29;22:53. doi: 10.1186/s13059-021-02269-3 (PMC7845113; doi:10.1186/s13059-021-02269-3)
Supplement: Supplementary file 1 — Additional file 1: Supplementary figure S1-S6. Figure S1. Diagram illustrating different types of nodetic versus edgetic perturbation leading to distinct phenotypes. Figure S2. Mutation rate is enriched in the ligand binding sites for these cancer types. Figure S3. Mutation rate is enriched in the phosphorylation sites for these cancer types. Figure S4. Correlation between the original results and new results by considering the amino acid specific background mutation rate and the amino acid composition for ligand-binding (LB) sites and post-translational modification (PTM) sites across 33 cancer types. Figure S5. Comparison of the results using different window sizes for the phosphorylation sites across all cancer types. Figure S6. Protein quantification is lowered in mutated phosphorylation sites than wild type sites in (A) breast invasive carcinoma, (B) colon adenocarcinoma, and (C) ovarian serous cystadenocarcinoma. [file 13059_2021_2269_MOESM1_ESM.pdf]

Fig. S1

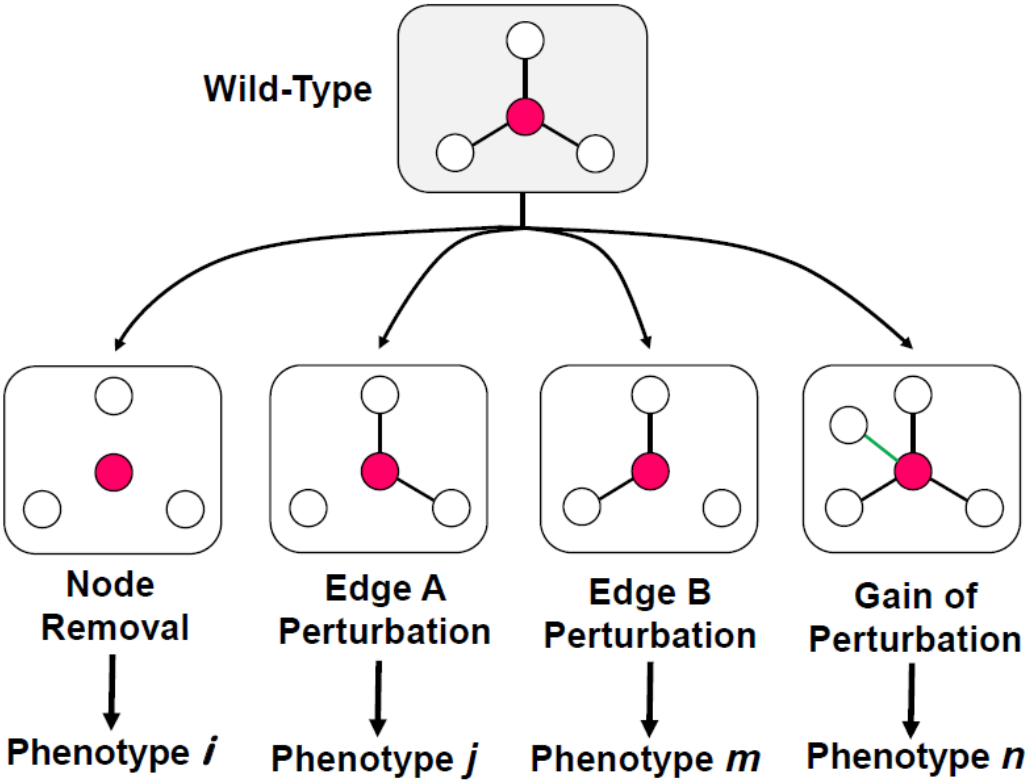

Fig. S1. Diagram illustrating different types of nodetic versus edgetic perturbation leading to distinct phenotypes. Nodetic effect refers to the effect that a mutation directly knockout or knockdown a gene, and consequently removing the protein and all its edges. Alternatively, mutation effects can also be protein-protein interaction (PPI) specific, causing removal or gain of specific PPIs, known as edgetic effect.

Fig. S2

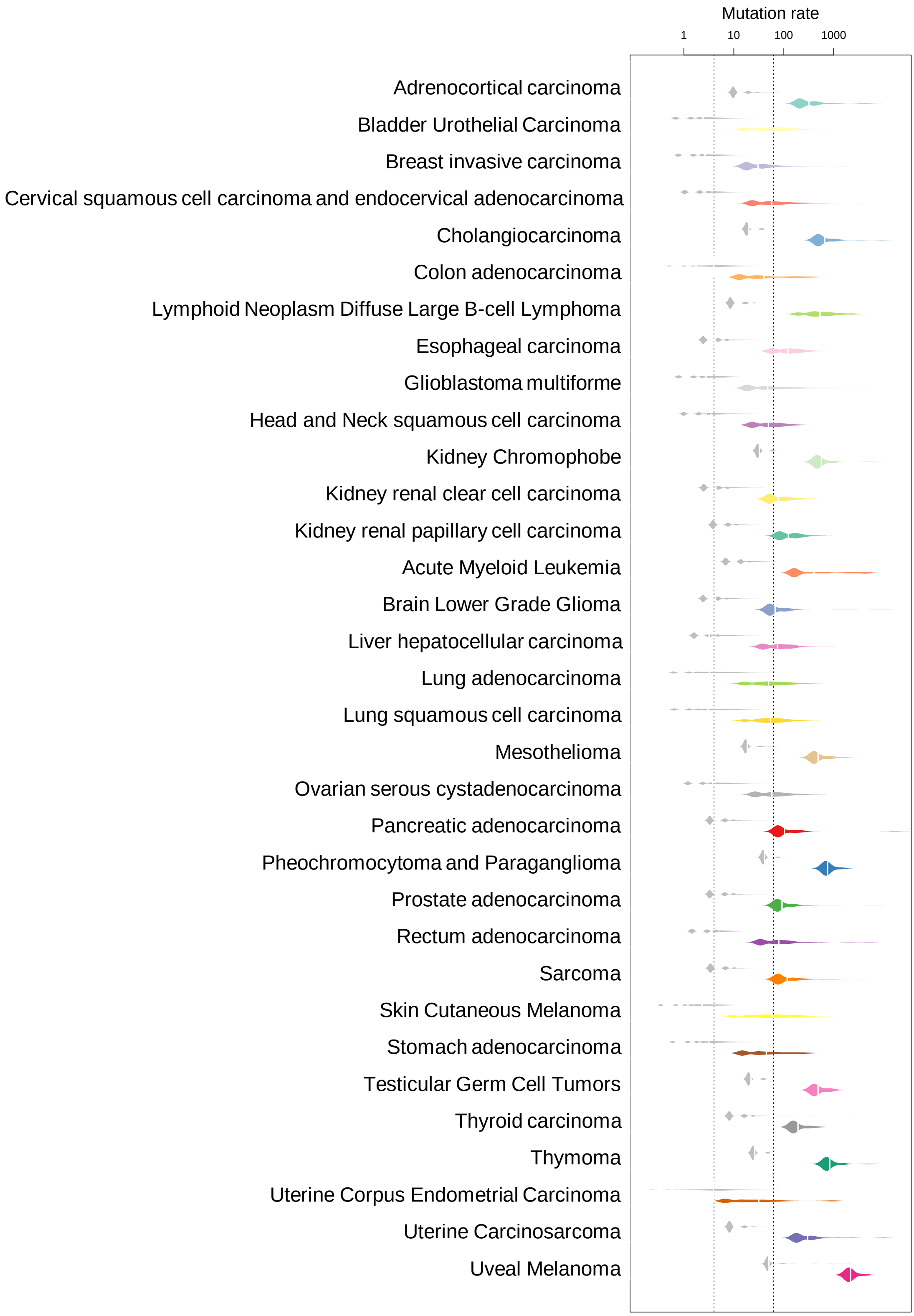

Fig. S2. Mutation rate is enriched in the ligand binding sites for these cancer types.

Fig. S3

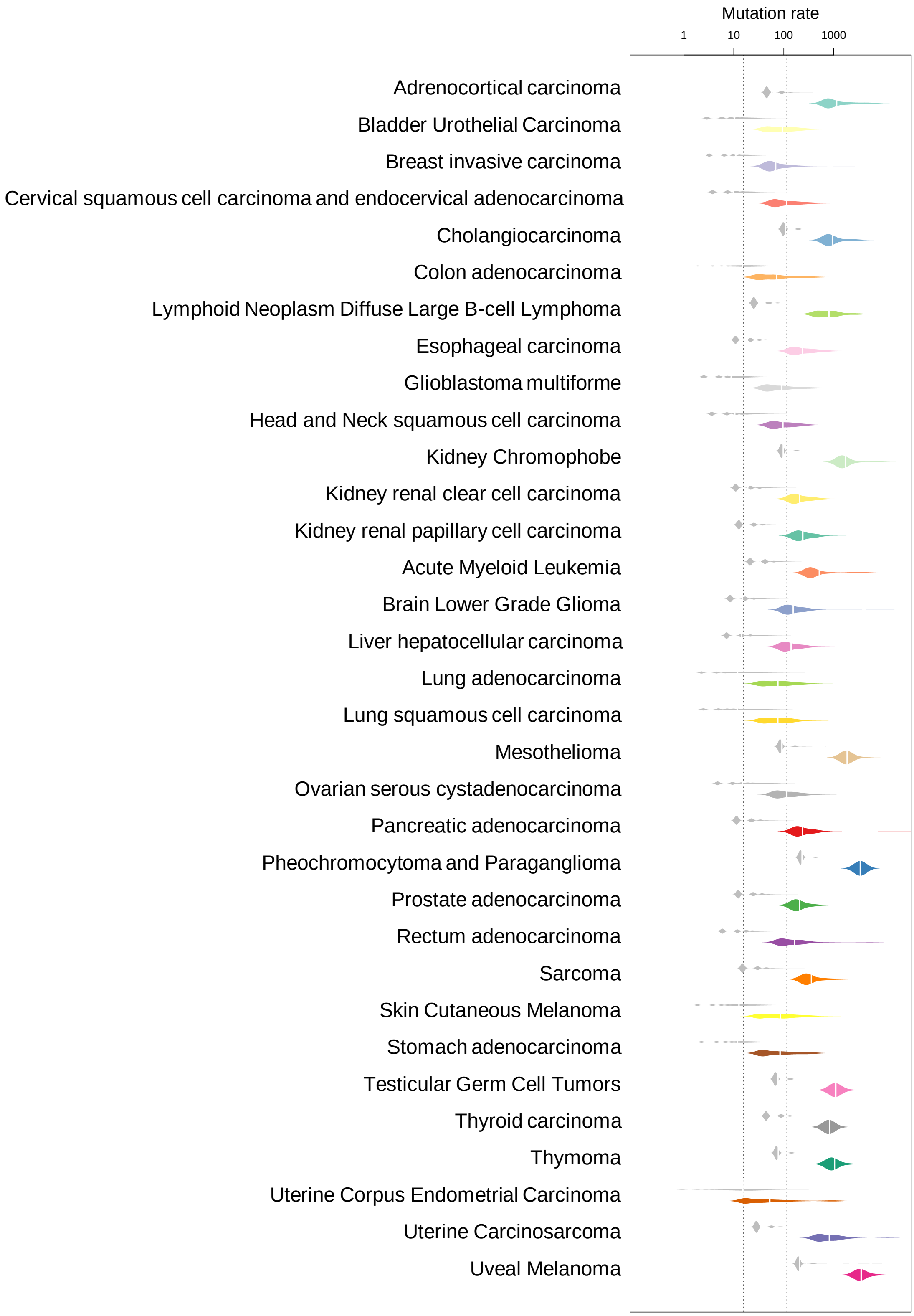

Fig. S3. Mutation rate is enriched in the phosphorylation sites for these cancer types.

Fig. S4

	LB	PTM
ACC	0.67	0.67
BLCA	0.80	0.75
BRCA	0.87	0.78
CESC	0.67	0.71
CHOL	0.71	0.77
COAD	0.69	0.79
DLBC	0.76	0.82
ESCA	0.84	0.78
GBM	0.70	0.78
HNSC	0.82	0.78
KICH	0.94	0.78
KIRC	0.93	0.80
KIRP	0.60	0.70
LAML	0.72	0.68
LGG	0.87	0.80
LIHC	0.86	0.79
LUAD	0.76	0.87

	LB	PTM
LUSC	0.80	0.86
MESO	0.73	0.77
OV	0.84	0.76
PAAD	0.91	0.82
PCPG	0.53	0.65
PRAD	0.84	0.71
READ	0.78	0.76
SARC	0.92	0.78
SKCM	0.79	0.89
STAD	0.71	0.78
TGCT	0.88	0.75
THCA	0.65	0.76
THYM	0.73	0.64
UCEC	0.73	0.88
UCS	0.84	0.82
UVM	0.97	0.75

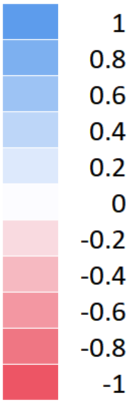

Fig. S4. Correlation between the original results and new results by considering the amino acid specific background mutation rate and the amino acid composition for ligand-binding (LB) sites and post-translational modification (PTM) sites across 33 cancer types.

ACC, Adrenocortical carcinoma. BLCA, Bladder Urothelial Carcinoma. BRCA, Breast invasive carcinoma. CESC, Cervical squamous cell carcinoma and endocervical adenocarcinoma. CHOL, Cholangiocarcinoma. COAD, Colon adenocarcinoma. DLBC, Lymphoid Neoplasm Diffuse Large B-cell Lymphoma. ESCA, Esophageal carcinoma. GBM, Glioblastoma multiforme. HNSC, Head and Neck squamous cell carcinoma. KICH, Kidney Chromophobe. KIRC, Kidney renal clear cell carcinoma. KIRP, Kidney renal papillary cell carcinoma. LAML, Acute Myeloid Leukemia. LGG, Brain Lower Grade Glioma. LIHC, Liver hepatocellular carcinoma. LUAD, Lung adenocarcinoma. LUSC, Lung squamous cell carcinoma. MESO, Mesothelioma. OV, Ovarian serous cystadenocarcinoma. PAAD, Pancreatic adenocarcinoma. PCPG, Pheochromocytoma and Paraganglioma. PRAD, Prostate adenocarcinoma. READ, Rectum adenocarcinoma. SARC, Sarcoma. SKCM, Skin Cutaneous Melanoma. STAD, Stomach adenocarcinoma. TGCT, Testicular Germ Cell Tumors. THCA, Thyroid carcinoma. THYM, Thymoma. UCEC, Uterine Corpus Endometrial Carcinoma. UCS, Uterine Carcinosarcoma. UVM, Uveal Melanoma.

Fig. S5

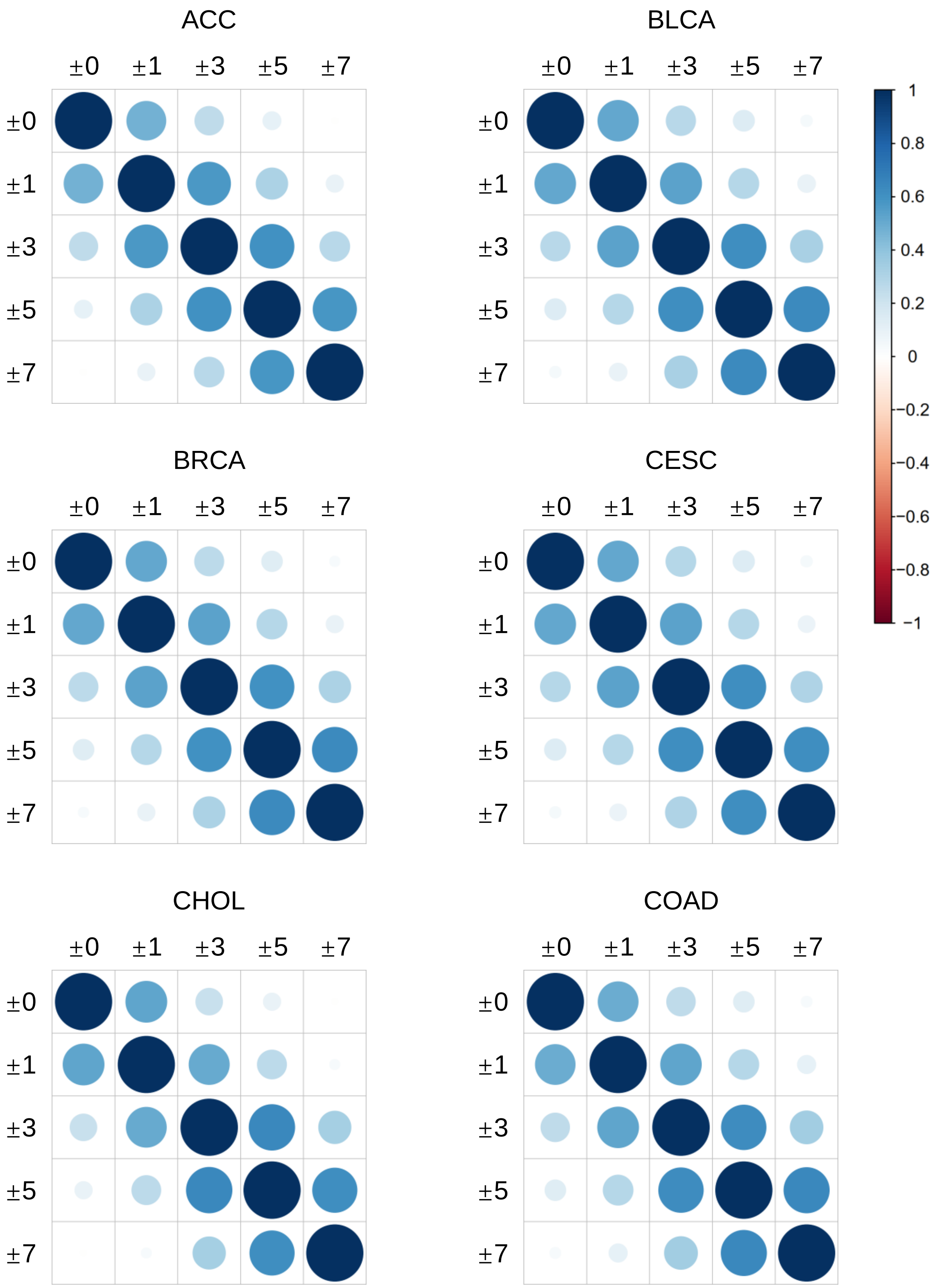

Fig. S5 continued

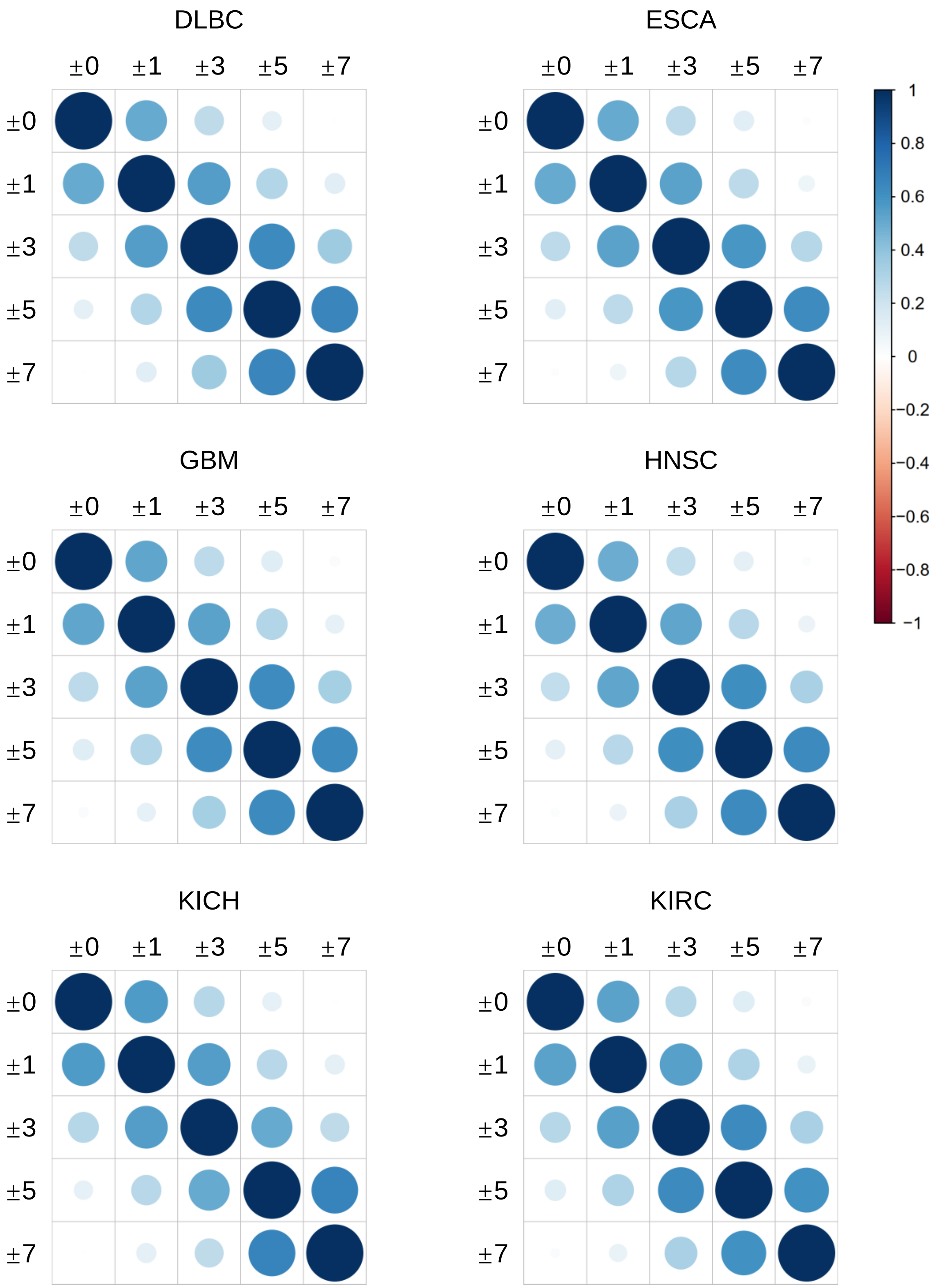

Fig. S5 continued

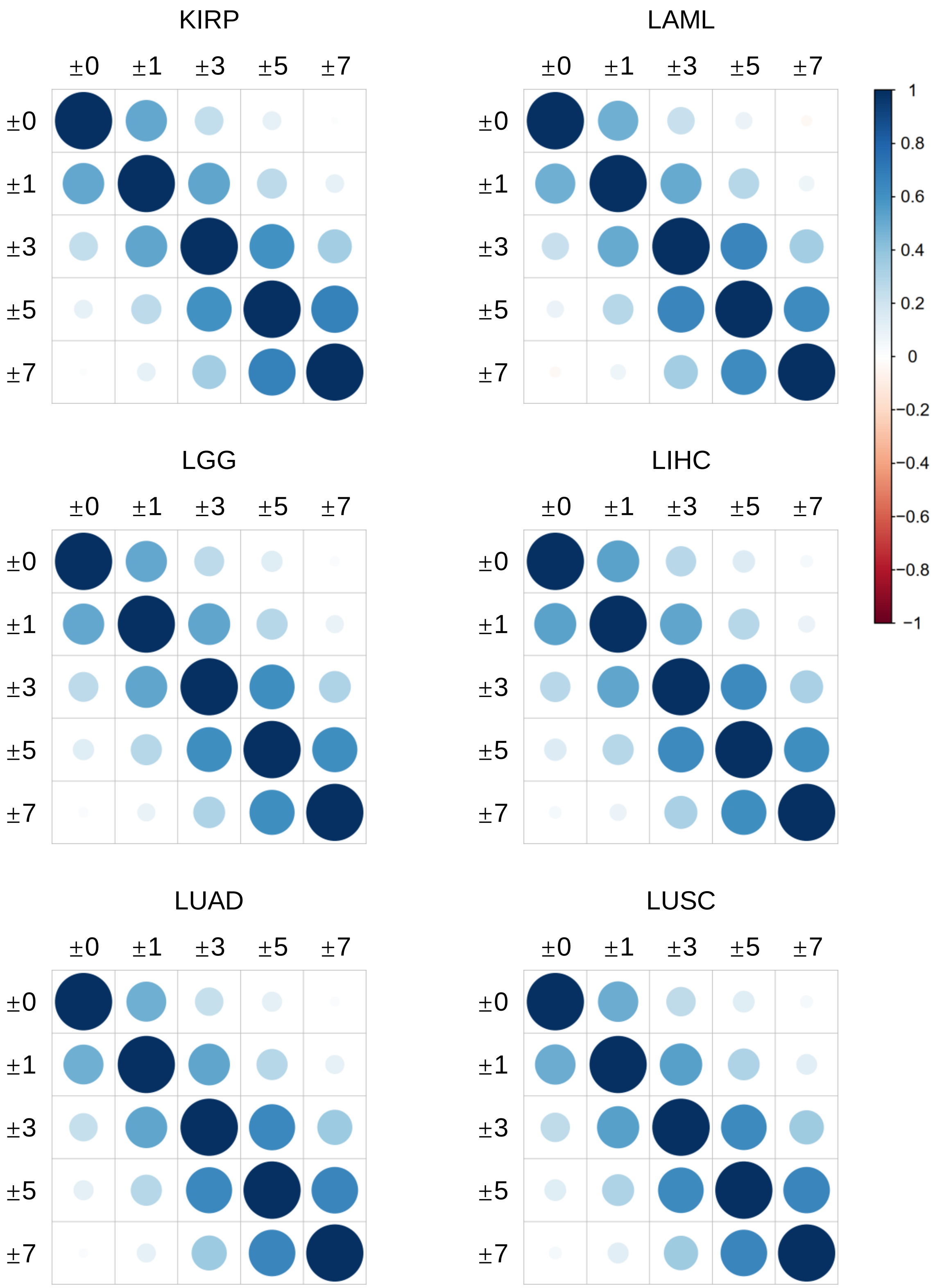

Fig. S5 continued

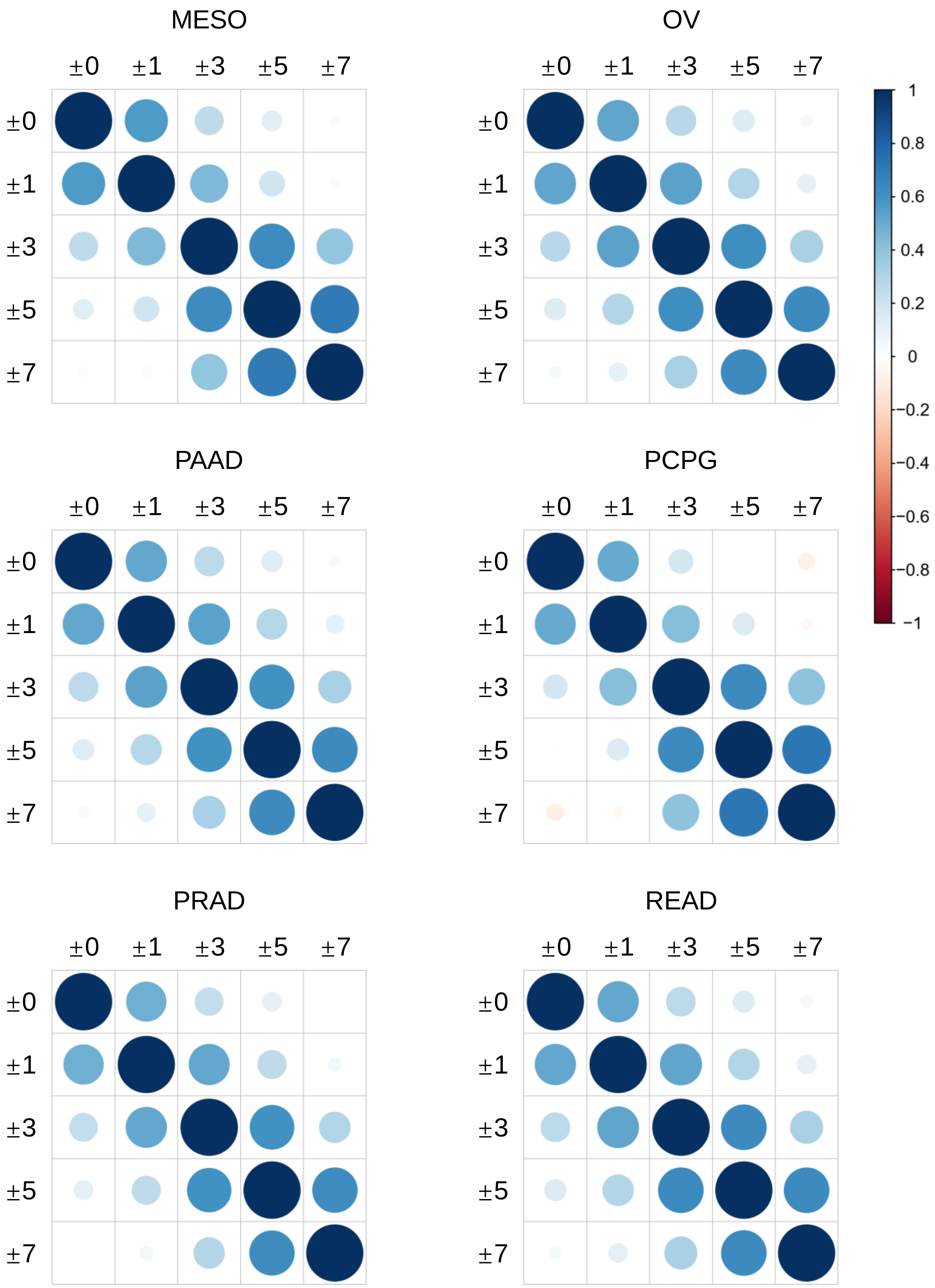

Fig. S5 continued

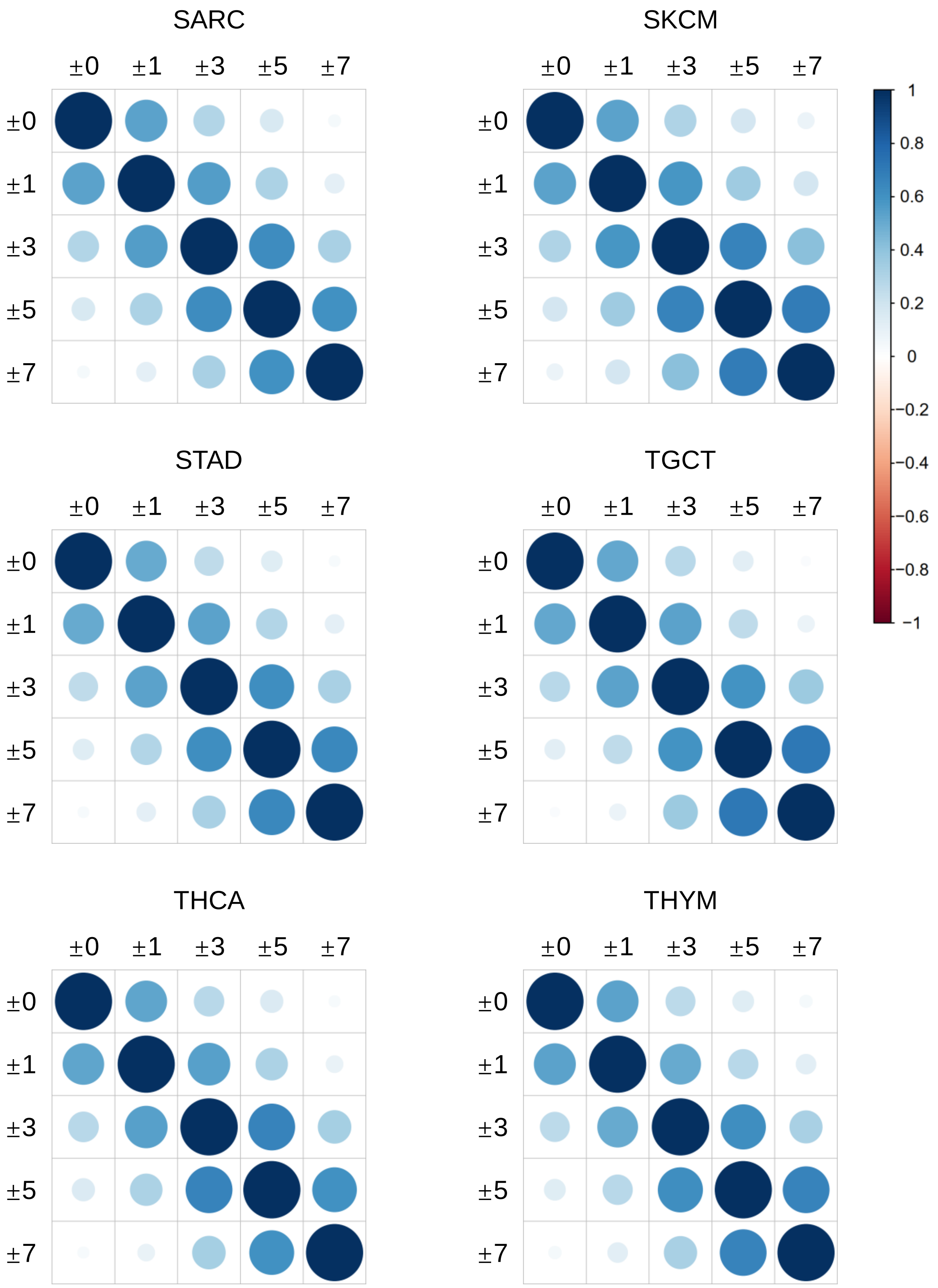

Fig. S5 continued

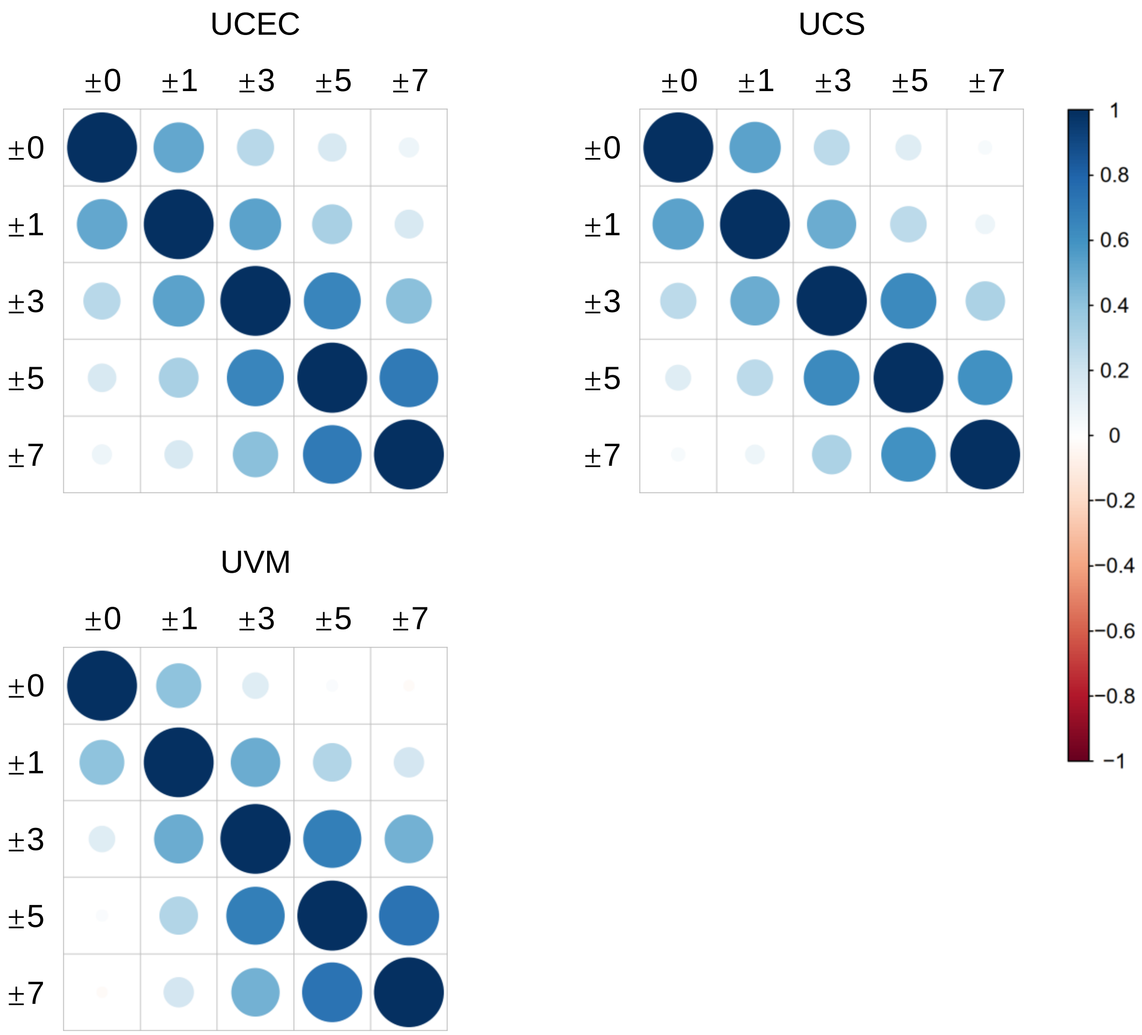

Fig. S5. Comparison of the results using different window sizes for the phosphorylation sites across all cancer types. We experimented with ±0 (exact site), ±3, ±5, and ±7, and found that the results of ±3, ±5, and ±7 to be highly correlated using spearman correlation.

ACC, Adrenocortical carcinoma. BLCA, Bladder Urothelial Carcinoma. BRCA, Breast invasive carcinoma. CESC, Cervical squamous cell carcinoma and endocervical adenocarcinoma. CHOL, Cholangiocarcinoma. COAD, Colon adenocarcinoma. DLBC, Lymphoid Neoplasm Diffuse Large B-cell Lymphoma. ESCA, Esophageal carcinoma. GBM, Glioblastoma multiforme. HNSC, Head and Neck squamous cell carcinoma. KICH, Kidney Chromophobe. KIRC, Kidney renal clear cell carcinoma. KIRP, Kidney renal papillary cell carcinoma. LAML, Acute Myeloid Leukemia. LGG, Brain Lower Grade Glioma. LIHC, Liver hepatocellular carcinoma. LUAD, Lung adenocarcinoma. LUSC, Lung squamous cell carcinoma. MESO, Mesothelioma. OV, Ovarian serous cystadenocarcinoma. PAAD, Pancreatic adenocarcinoma. PCPG, Pheochromocytoma and Paraganglioma. PRAD, Prostate adenocarcinoma. READ, Rectum adenocarcinoma. SARC, Sarcoma. SKCM, Skin Cutaneous Melanoma. STAD, Stomach adenocarcinoma. TGCT, Testicular Germ Cell Tumors. THCA, Thyroid carcinoma. THYM, Thymoma. UCEC, Uterine Corpus Endometrial Carcinoma. UCS, Uterine Carcinosarcoma. UVM, Uveal Melanoma.

Fig. S6

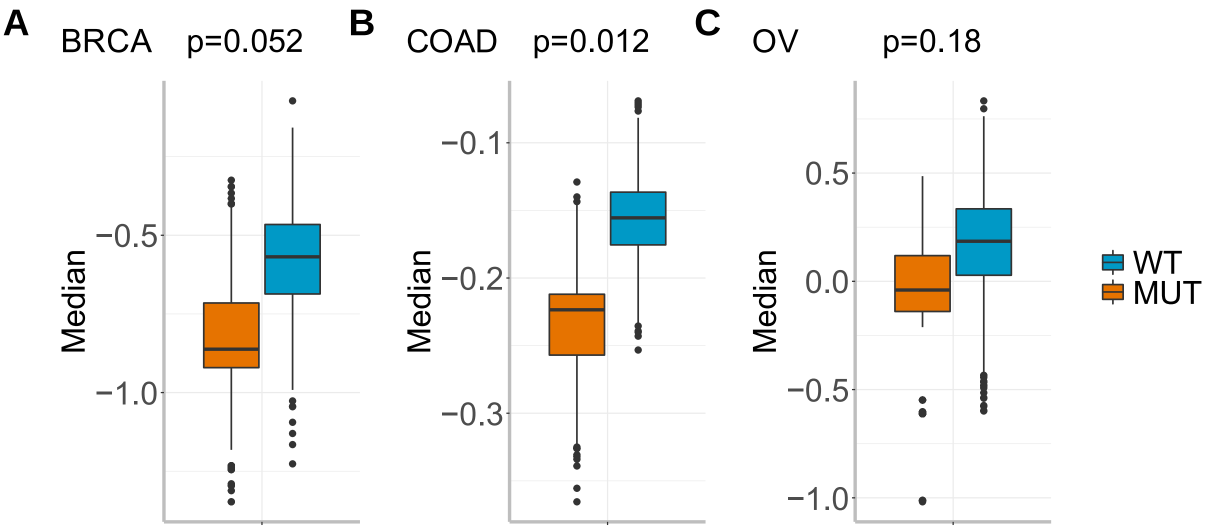

Fig. S6. Protein quantification is lowered in mutated phosphorylation sites than wild type sites in (**A**) breast invasive carcinoma (n=54), (**B**) colon adenocarcinoma (n=486), and (**C**) ovarian serous cystadenocarcinoma (n=10). We downloaded the site level phosphoproteome data from http://www.linkedomics.org. The data were divided into two groups: mutated phosphosites and wild type phosphosites. We then compared the expression levels of these two groups. The final distributions in the boxplots were derived from resampling.
