# Supplementary material for: My personal mutanome: a computational genomic medicine platform for searching network perturbing alleles linking genotype to phenotype
Source: Genome Biol. 2021 Jan 29;22:53. doi: 10.1186/s13059-021-02269-3 (PMC7845113; doi:10.1186/s13059-021-02269-3)
Supplement: Supplementary file 2 — Additional file 2. Review history. [file 13059_2021_2269_MOESM2_ESM.docx]

Review History

**First round of review**

**Reviewer 1**

**Are you able to assess all statistics in the manuscript, including the appropriateness of statistical tests used?** Yes

**Comments to author:**

the study "My Personal Mutanome: A Personalized Cancer Medicine Platform for Searching Network Perturbing Alleles Linking Somatic Genotype to Phenotype", Zhou et al. developed a useful database, My Personal Mutanome (MPM), to facilitate cancer personalized medicine via protein mutation. They also included protein-protein interaction (PPI) network clinical information including survival and drug response for these mutated samples. They database is overall comprehensive and useful.

1. It looks like that authors mapped those mutated sites to known PPI network to infer the potential alternation. Is it possible that mutation sites are involved in unknown PPI which is not included in known resource? In addition, for all mutation associated PPI, is it possible to know the causal relationships? The authors may at least discuss these important questions.

2. Could the authors introduce the definition and functional role of "nodetic" and "edgetic" in more details?

3. The authors should add more information, including functional modules, usage and explanation of output in the about page, which will help users to query and understand output conveniently.

4. For drug response part, the reviewer suggests authors using adjust p value instead of original p value to narrow down the false positive rate. For example, Figure 5G, although the p value is 0.04, the distribution of IC50 in WT and MUT group are very similar. In addition, sample size for both WT and MUT group should be clarified.

**Reviewer 2**

**Are you able to assess all statistics in the manuscript, including the appropriateness of statistical tests used?** No

**Comments to author:**

Zhou et al provide a resource generated from integration of somatic missense mutations with annotations of PPI network and protein functional sites to facilitate identification of clinically actionable mutations. They also correlate pan-cancer survival data and drug response data from cell lines with the presence or absence of these mutation to identify therapeutic potential in a personalised medicine approach.

The overall implementation of the web based resource is good and integration of complementary annotations particularly in the context of PPI networks or protein functional sites is useful, however, the authors fail to demonstrate the utility of this resource beyond two simple examples.

Here are some major changes the authors may wish to consider:

1. A major issue with this resource is that the manuscript lacks any evaluation and analysis on the features for which data is generated.

For the nodetic effects, the authors infer the impact of mutations on protein functional sites. So a basic expectation would be to see whether there is an enrichment of missense mutations in protein functional sites in their analysis. Similarly, it is unclear whether the presence of the mutation may have a functional effect. In this context one would expect that disruption of a phosphosite should lead to loss of signalling. Since a lot of phosphorus-proteomics data is now available for a subset of TCGA tumour types, one would expect to see differences in the phosphorylation status based on the presence or absence of a mutation.

Similarly, edgetic effect should rewire protein interaction networks and it is not clear whether the metric used to identify edgetic effects actually works and whether it is able to identify reliable examples edgetic effects. Systematic benchmarking and analysis of SMEs should be performed to show which potential edges are disrupted in PPIs.

2. Another aspect that is completely lacking is new and interesting biology or novel targets from the analysis of nodetic and edgetic effect of mutations.

3. Why the sequence composition was not accounted for while calculating the background mutation rate at protein interfaces and functional sites?

4. Not all missense mutations are pathogenic and impact of mutations at protein interfaces can be evaluated using tools such as FOLDX. Similarly, protein sequence conservation can be a determinant of the likelihood of a somatic mutation to be pathogenic. These information could be potentially useful additions to the database.

5. The database would have been more exciting had it offered the possibility to upload a vcf file of somatic mutations which could then be mapped to identify 'nodetic' and 'edgetic' effects in a sample. Without this functionality, the existing name of the database (MPM) is misleading and a more appropriate title should be considered.

Minor Point:

1. Grammatical Typos within figure 2:

Q7. What mutations can potentially affect them,

Q8. Does the cell line with the mutated functional site react differently to drugs.

2. Line 11 in Introduction has grammatical mistake.

Overall I think the current version of the manuscript lacks biological insights and a comprehensive analysis/evaluation of the resource is highly desirable.

**Reviewer 3**

**Are you able to assess all statistics in the manuscript, including the appropriateness of statistical tests used?** No

**Comments to author:**

In the manuscript "My Personal Mutanome: A Personalized Cancer Medicine Platform for Searching Network Perturbing Alleles Linking Somatic Genotype to Phenotype" Zhou and colleagues describe a new database aiming to link an individual's genetic makeup to the phenotype they manifest. The authors have undoubtedly hit upon a critical question as the endlessly expanding glut of genomic sequence data has failed to result in dramatic improvements of patient outcomes, it is unclear if "My Personal Mutanome" has bridged this gap.

The first concern for any model or dataset lies in the fundamental input data upon which it is based. The authors integrated an expansive set of data encompassing ~500,000 somatic mutations, over 120,000 physical PPIs, and over 500,000 functional sites. The risk with such large datasets is that they are often (1) intrinsically biased due to sociological and funding prioritization, (2) of vastly differing quality depending on the methods and rigor applied to generate the data and (3) massively incomplete. It is unclear if the authors explored any of these potential issues or attempted to control for them.

To identify interaction interfaces the authors used three very different sources of data, PDB, ÉCLAIR, and Interactome3D. While PDB contains experimentally derived structural data of the proteins, including in some cases the pair of interacting proteins in question, ECLAIR (which contributed the vast majority of the interface data) uses a machine learning approach to try to predict the interface between two proteins. Such approaches are fraught with problems in particular for protein lacking clear homologues or poorly studied proteins. It would be much more convincing to see how the model could perform with "gold standard" interface data from 3D models and then determining if predicted interface data such as that from ÉCLAIR could match the performance of the 3D models. What would be the impact of only using data from the resolved 3D models?

To evaluate the impact of a variant on a functional site the authors looked for mutations with 7 amino acids (upstream or downstream) of the functional site. It is unclear how they settled on this 15 amino-acid window or if they considered alternative window sizes. While clearly the amino acid sequence surrounding a site can impact the accessibility of a site, it is hard to believe that this is equal for all types of sites or all proteins. Did the authors attempt to verify the impact of any mutations flanking a functional site identified as potentially critical in their analysis? It would be relatively simple to demonstrate the impact of mutations surrounding a functional (e.g. phosphorylation) site using conventional biochemical approaches.

Similarly, the authors look for an enrichment in mutations in the region identified to mediate protein-protein interactions. Did the authors test any of these interactions? Firstly, to demonstrate that they had correctly identified the interface region, and secondly that the mutations did indeed impact the interaction? Without such evidence it is hard to support the authors conclusion that these mutations are perturbing interactions and hence contributing to the observed phenotype.

For the drug responses, it is again unclear from the text how the authors associated a drug to a gene, or if they validated any of the connections they proposed. Can the authors provide either experimental data to show that the drug impact the gene they have mapped it to, or develop some sort of gold-standard reference set (not used in the training) and show how often they correctly paired drugs with target genes.

In summary, while there is undoubtedly a compelling need for better tools to functionalize genomic data, it is unclear if the authors have succeeded in meeting this goal. The methods used to select the data to incorporate in the analysis and how the authors showed that these dataset were the best to use are unclear. Better control using training and test sets to demonstrate the accuracy of their tool would go a long way to convincing the reader that this is a valuable and useful resource.

Minor

HGNC reports 19,324 protein coding genes, yet the authors describe "21,759 proteins" (page 10 line 21). Could the authors clarify what they are considering as a unique protein in their study?

**Authors Response**

**Point-by-point responses to the reviewers’ comments:**

**Responses to Reviewer #1:**

In the study "My Personal Mutanome: A Personalized Cancer Medicine Platform for Searching Network Perturbing Alleles Linking Somatic Genotype to Phenotype", Zhou et al. developed a useful database, My Personal Mutanome (MPM), to facilitate cancer personalized medicine via protein mutation. They also included protein-protein interaction (PPI) network clinical information including survival and drug response for these mutated samples. They database is overall comprehensive and useful. **Response:** We thank the Reviewer for this kind summary, and the overall constructive feedback regarding our study.

1. It looks like that authors mapped those mutated sites to known PPI network to infer the potential alternation. Is it possible that mutation sites are involved in unknown PPI which is not included in known resource?

**Response:** Yes. It is possible that the mutations are involved in unknown PPIs owing to data incompleteness issue. Although we reconstructed a comprehensive human interactome by assembling multiple types of experimental evidences based on our sizeable efforts, the PPI networks we used may be still incomplete. Therefore, we will keep updating our human protein-protein interactome in our future studies. We have discussed the incompleteness issue of the human interactome in the Discussion section.

2. In addition, for all mutation associated PPI, is it possible to know the causal relationships? The authors may at least discuss these important questions.

**Response:** We thank the Reviewer for this important point. Yes, our recent study (Cheng et al., bioRxiv 2020, under minor revision by *Nature Genetics*) demonstrated that PPI interface mutations can provide likely causal relationships in human diseases including cancer, and we validated it experimentally. For example, we experimentally validated the network effects of 13 PPI mutations using a systematic, binary yeast two- hybrid interaction assay (Cheng et al., bioRxiv 2020). We further showed that ALOX5 p.Met146Lys at the ALOX5-MAD1L1 interface and RXRA p.Ser427Phe at the RXRA-PPARG interface promote significant tumor cell growth using cell line-based functional assays, providing a proof-of-concept of causal relationships of PPI interface mutations. We have added these details in the revised manuscript (page 5).

**Reference**

1. Cheng F, Zhao J, Wang Y, Lu W, Liu Z, Zhou Y, et al. Comprehensive characterization of protein-protein interaction network perturbations by human disease mutations. doi: <https://doi.org/10.1101/2020.09.18.302588>

**3.** Could the authors introduce the definition and functional role of "nodetic" and "edgetic" in more details?

**Response:** Node and edge refer to the elements that comprise the protein-protein interaction network. As shown in **Figure 1** and the newly added **Figure S1** (**Extended Figure 1**), nodes are the proteins and edges are the physical interactions among them. Both nodes (proteins) and edges (PPIs) can be affected by mutations. Mutations can cause nodetic and edgetic effects (Vidal et al., *Cell* 2011; Sahni et al., *Curr Opin Genet Dev.* 2013; Sahni et al., *Cell* 2015). Nodetic effect refers to the effect that a mutation directly knockout or knockdown a gene/protein, and consequently removing the protein and all its edges. Alternatively, mutation effects can also be PPI specific, causing removal or gain of specific PPIs, known as edgetic effects. We have added the clearer definition for both "nodetic" and "edgetic" in the revised manuscript (page 5).

**Extended Figure 1.** Diagram illustrating different types of nodetic versus edgetic perturbation leading to distinct diseases/phenotypes in the human protein-protein interactome networks.

**References**

1. Vidal M, Cusick ME, Barabási AL. Interactome networks and human disease. Cell. 2011;144:986–98.
2. Sahni N, Yi S, Zhong Q, Jailkhani N, Charloteaux B, Cusick ME, et al. Edgotype : the link between genotype and phenotype. Curr Opin Genet Dev. 2013;23:649–57.
3. Sahni N, Yi S, Taipale M, Fuxman Bass JI, Coulombe-Huntington J, Yang F, et al. Widespread macromolecular interaction perturbations in human genetic disorders. Cell. 2015;161:647–60.
4. The authors should add more information, including functional modules, usage and explanation of output in the about page, which will help users to query and understand output conveniently.

**Response:** We have added a new “Help” page that provides the detailed functional modules, usage and explanation of output in our database.

5. For drug response part, the reviewer suggests authors using adjust p value instead of original p value to narrow down the false positive rate. For example, Figure 5G,

although the p value is 0.04, the distribution of IC50 in WT and MUT group are very similar. In addition, sample size for both WT and MUT group should be clarified. **Response:** In our updated database, we have added the adjusted p-values as well as the sample size for the WT and MUT groups. For Figure 5G, our aim is to illustrate how the users can use our database to explore drug responses altered by PPI interface mutations. We have added adjusted p-value in the revised Figure 5G as well.

**Responses to Reviewer #2:**

Zhou et al provide a resource generated from integration of somatic missense mutations with annotations of PPI network and protein functional sites to facilitate identification of clinically actionable mutations. They also correlate pan-cancer survival data and drug response data from cell lines with the presence or absence of these mutation to identify therapeutic potential in a personalised medicine approach. The overall implementation of the web-based resource is good and integration of complementary annotations particularly in the context of PPI networks or protein functional sites is useful, however, the authors fail to demonstrate the utility of this resource beyond two simple examples. **Response:** We thank the Reviewer for the overall constructive feedback regarding our study. In the past 2 months, we have performed extensive additional computational analysis and new experimental data to improve the utility of our “My Personal Mutanome” tools in multiple ways. The new data we now incorporated in the revised version of the manuscript are attached as below to demonstrate the utility of our database.

1. A major issue with this resource is that the manuscript lacks any evaluation and analysis on the features for which data is generated.

**Response:** We have performed extensive new computational analyses and experiments to intensify the rigor and reproducibility of our presented tool, termed “My Personal Mutanome”. The new computational and experimental data we now incorporated are attached as below and added in the revised manuscript as well.

2. For the nodetic effects, the authors infer the impact of mutations on protein functional sites. So a basic expectation would be to see whether there is an enrichment of missense mutations in protein functional sites in their analysis.

**Response:** We thank the Reviewer for this important point. We have extensively demonstrated that somatic (missense) mutations were significantly enriched in functional sites of proteins: (1) ligand-binding sites (Zhao et al., *Mol Cell Proteomics* 2016) [original reference 12], (2) allosteric sites (Shen et al., *Am J Hu Genetics* 2017) [original reference 13], (3) phosphorylation sites (Zhao et al., *Cancer Research* 2018) [original reference 14], and (4) PPI interfaces (Cheng et al., bioRxiv 2020). To respond the Reviewer’s concern, we re-performed new enrichment analyses for over 10,000 tumor exomes across 33 cancer types. Consistent with our previous studies, somatic (missense) mutations are significantly enriched in the protein-ligand binding sites (**Figure S2**, **Extended Figure 2**) and phosphorylation sites (**Figure S3**, **Extended Figure 3**) compared to non-ligand binding and non-phosphorylation sites, respectively. We have added new findings and more explanations in the revised manuscript.

Mutation rate

1	10	100	1000

**Extended Figure 2.** Somatic missense mutations are significantly enriched in the ligand binding sites (color bin) compared to non-ligand-binding regions (grey bin) across all 33

cancer types (P < 2.2×10^-16^, two-sided Wilcox test). Mutation rate is quantified by per 1 million residues.

Mutation rate

1	10	100	1000

**Extended Figure 3.** Somatic missense mutations are significantly enriched in the phosphorylation sites (color bin) compared to non-phosphorylation sites (grey bin) across all 33 cancer types (P < 2.2×10^-16^, two-sided Wilcox test). Mutation rate is quantified by per 1 million residues.

**References**

1. Zhao J, Cheng F, Wang Y, Arteaga CL, Zhao Z. Systematic prioritization of druggable mutations in ~5000 genomes across 16 cancer types using a structural genomics-based approach. Mol Cell Proteomics. 2016;15:642–56.
2. Shen Q, Cheng F, Song H, Lu W, Zhao J, An X, et al. Proteome-Scale Investigation of Protein Allosteric Regulation Perturbed by Somatic Mutations in 7,000 Cancer Genomes. Am J Hum Genet. 2017;100:5–20.
3. Zhao J, Cheng F, Zhao Z. Tissue-specific signaling networks rewired by major somatic mutations in human cancer revealed by proteome-wide discovery. Cancer Res. 2017;77:2810–21.
4. Cheng F, Zhao J, Wang Y, Lu W, Liu Z, Zhou Y, et al. Comprehensive characterization of protein-protein interaction network perturbations by human disease mutations. bioRxiv. doi: <https://doi.org/10.1101/2020.09.18.302588>

**3.** Similarly, it is unclear whether the presence of the mutation may have a functional effect. In this context one would expect that disruption of a phosphosite should lead to loss of signalling. Since a lot of phosphorus-proteomics data is now available for a subset of TCGA tumour types, one would expect to see differences in the phosphorylation status based on the presence or absence of a mutation.

**Response:** We thank the Reviewer for this great point. We agree with the Reviewer that there are some available phosphorus-proteomics data or proteogenomics data

resources, such as The National Cancer Institute's Clinical Proteomic Tumor Analysis Consortium (CPTAC). However, most CPTAC datasets for TCGA tumour types have been postponed for publication purposes. We thus only selected COAD (colon cancer) without publication restriction. We analyzed COAD phosphorus-proteomics data and did find relatively lower protein quantification in mutated phosphorylation sites than wild type sites (**Figure S6**, **Extended Figure 4**), suggesting loss of signaling by somatic missense mutations.

**Extended Figure 4.** Protein quantification in COAD patients with mutated phosphorylation sites is lower than patients with wild type phosphorylation sites. Phosphoproteomics data of mutated phosphorylation sites were compared to wild type sites. Resampling was used to generate the respective distribution.

**4.** Similarly, edgetic effect should rewire protein interaction networks and it is not clear whether the metric used to identify edgetic effects actually works and whether it is able to identify reliable examples edgetic effects. Systematic benchmarking and analysis of SMEs should be performed to show which potential edges are disrupted in PPIs. **Response:** We thank the Reviewer for this critique. We apologized that we didn’t provide systematic benchmarking validation data from our recent bioRxiv paper in our original manuscript: (1) Disease-associated germline mutations are significantly enriched in sequences encoding protein-protein interfaces compared to mutations identified in healthy subjects from the 1000 Genomes and ExAC projects: (2) Somatic missense mutations are significantly enriched in PPI interfaces compared to non-interfaces in 10,861 human exomes across 33 cancer subtypes/types from The Cancer Genome Atlas (P < 2.2×10^-16^, two-sided Wilcox test, **Extended Figure 5a**); (3)

Deleterious substitutions identified by SIFT and PolyPhen scores are significantly enriched in the PPI interfaces (**Extended Figure 5b, c**); (4) We experimentally validated the network effects of 13 oncoPPIs using a systematic binary interaction assay; and (5) We further showed that ALOX5 p.Met146Lys at the ALOX5-MAD1L1 interface and RXRA p.Ser427Phe at the RXRA-PPARG interface promote significant tumor cell growth using cell line-based functional assays. In summary, these comprehensive benchmarking validation data offers a proof-of-concept of “edgetic” effects of PPI interface mutations.

**Extended Figure 5.** Characterization of PPI interface mutations. (a) somatic missense mutations are enriched in the PPI interfaces compared to non-interfaces. (b, c) deleterious substitutions identified by SIFT and PolyPhen scores are significantly enriched in the PPI interfaces. Source: Cheng et al., bioRxiv 2020 Figure 2.

**Reference**

1. Cheng F, Zhao J, Wang Y, Lu W, Liu Z, Zhou Y, et al. Comprehensive characterization of protein-protein interaction network perturbations by human disease mutations. bioRxiv. doi: <https://doi.org/10.1101/2020.09.18.302588> **5.** Another aspect that is completely lacking is new and interesting biology or novel targets from the analysis of nodetic and edgetic effect of mutations.

**Response:** We apologized that we didn’t provide the detailed biological insights in our original manuscript as we submitted our manuscript as Database paper, not original Research article. As stated in *Genome Biology* guideline: “*This is our format for publishing database with major utility to a broad field of research, but that* ***do not provide significant biological insights***.” Please see editor’s comment in the end of this letter.

Our team focused on “nodetic” and “edgetic” effects of somatic mutations, a key cancer systems biology research field for almost one decade. For example, we first showed the network-perturbing somatic mutations in tumorigenesis and tumor evolution in 2014 (Cheng et al., *Mol Biol Evol* 2014). In the past 5 years, we have demonstrated that nodetic and edgetic effects provide important insights into functional consequences of somatic mutations for human cancer and offer useful tools to identify likely driver genes for tumorigenesis and anticancer drug responses (Zhao et al., *Mol Cell Proteomics* 2016; Shen et al., *Am J Hu Genetics* 2017; Zhao et al., *Cancer Research* 2018; Cheng et al., bioRxiv 2020). For example, we identified multiple potential driver mutations/genes and validated them experimentally: (1) using allosteric mutation model (Shen et al., *Am J Hu Genetics* 2017), we experimentally confirmed that p.Pro360Ala on PDE10A played a potential oncogenic role in mediating tumorigenesis in non-small cell lung cancer (NSCLC); (2) In our recent bioRxiv paper, we experimentally validated the network effects of 13 oncoPPIs using a systematic binary interaction assay; and (3) We further showed that ALOX5 p.Met146Lys at the ALOX5-MAD1L1 interface and RXRA p.Ser427Phe at the RXRA-PPARG interface promote significant tumor cell growth using cell line-based functional assays.

In summary, “My Personal Mutanome” that contains the comprehensive analyses of nodetic and edgetic effects of disease mutations offers a useful cancer systems biology tool for accelerating development of precision cancer medicine with a novel explanatory mechanism. We have added more explanations in the revised manuscript.

**References**

1. Cheng F, Jia P, Wang Q, Lin CC, Li WH, Zhao Z. Studying tumorigenesis through network evolution and somatic mutational perturbations in the cancer interactome. Mol Biol Evol. 2014;31:2156–69.
2. Zhao J, Cheng F, Wang Y, Arteaga CL, Zhao Z. Systematic prioritization of druggable mutations in ~5000 genomes across 16 cancer types using a structural genomics-based approach. Mol Cell Proteomics. 2016;15:642–56.
3. Shen Q, Cheng F, Song H, Lu W, Zhao J, An X, et al. Proteome-Scale Investigation of Protein Allosteric Regulation Perturbed by Somatic Mutations in 7,000 Cancer Genomes. Am J Hum Genet. 2017;100:5–20.
4. Zhao J, Cheng F, Zhao Z. Tissue-specific signaling networks rewired by major somatic mutations in human cancer revealed by proteome-wide discovery. Cancer Res. 2017;77:2810–21.
5. Cheng F, Zhao J, Wang Y, Lu W, Liu Z, Zhou Y, et al. Comprehensive characterization of protein-protein interaction network perturbations by human disease mutations. bioRxiv. doi: <https://doi.org/10.1101/2020.09.18.302588>
6. Why the sequence composition was not accounted for while calculating the background mutation rate at protein interfaces and functional sites?

**Response:** To answer this question, we performed the following new experiments. We considered the sequence composition by computing amino acid specific background mutation rate and considering the amino acid composition around each ligand binding site or PTM site. When comparing the result from this new analysis with our original findings without considering sequence composition, we found these two results are highly correlated across 33 cancer types (**Figure S4**, **Extended Figure 6**). To keep consistent with our previous studies (Zhao et al., *Mol Cell Proteomics* 2016; Shen et al., *Am J Hu Genetics* 2017; Zhao et al., *Cancer Research* 2018; Cheng et al., bioRxiv 2020), we prefer not add new results in the current manuscript. Yet, your suggestions will motivate our future studies.

**Extended Figure 6.** Spearman correlation between the original results and new results by considering the amino acid specific background mutation rate and the amino acid

composition for **ligand-binding** (LB) sites and **post-translational modification** (PTM) sites across 33 cancer types. Correlation coefficient was computed by using the p value vector (-log10 transformed) of all the mutated LB sites or PTM sites from significance test.

7. Not all missense mutations are pathogenic and impact of mutations at protein interfaces can be evaluated using tools such as FOLDX. Similarly, protein sequence conservation can be a determinant of the likelihood of a somatic mutation to be pathogenic. These information could be potentially useful additions to the database. **Response:** We thank the Reviewer for this great point. FOLDX is a protein structure-based webserver and we cannot get results from FOLDX for ~500K mutations in a short period due to high computational cost of FOLDX. In addition, FOLDX cannot provide high-confidence score for a large amount of mutations without known protein structure data. We therefore added another two commonly used scores, SIFT score and PolyPhen score, for all curated mutations in our database.
8. The database would have been more exciting had it offered the possibility to upload a vcf file of somatic mutations which could then be mapped to identify 'nodetic' and 'edgetic' effects in a sample. Without this functionality, the existing name of the database (MPM) is misleading and a more appropriate title should be considered. **Response:** We thank the Reviewer for this great suggestion. Due to very strict restriction on patient data safety implemented by our current institute, Cleveland Clinic, we are not allowed to implement a web server which provides automatic calculations for the external users outside of Cleveland Clinic healthcare systems. We therefore only implement the database query functions in our new database. To accommodate for this limitation, we uploaded all codes for the mutation mapping pipeline we have used to GitHub (<https://github.com/ChengF-Lab/mutanome)>. In addition, we have added a new search function where the user can input a list of mutations and retrieve a summary of output (**Extended Figure 7**).

**Extended Figure 7.** User can now input a list of mutations and retrieve a summary of them. The summary includes the SIFT and PolyPhen scores and mutant cases in TCGA. User can click a mutation to load more information from this table.

Minor Point:

**1.** Grammatical Typos within figure 2:

Q7. What mutations can potentially affect them.
Q8. Does the cell line with the mutated functional site react differently to drugs. **Response:** Thank you for pointing out the grammatical mistakes and typos. We have updated the webpage and updated Figure 2 as suggested.

**2.** Line 11 in Introduction has grammatical mistake.

**Response:** English was double checked and polished by native speakers of our co-

authors, including Charis Eng and Tim Chan, in the revised manuscript.

Overall I think the current version of the manuscript lacks biological insights and a comprehensive analysis/evaluation of the resource is highly desirable.

**Response:** We thank the Reviewer for this critique. As stated by Editor in the end of the letter: “*This is our format for publishing database with major utility to a broad field of research, but that* ***do not provide significant biological insights***.” for database article. However, we have performed extensive additional computational analyses and added new computational and experimental findings in the revised manuscript. Please see more detailed responses in Comments #2-5.

**Responses to Reviewer #3:**

1. In the manuscript "My Personal Mutanome: A Personalized Cancer Medicine

Platform for Searching Network Perturbing Alleles Linking Somatic Genotype to Phenotype" Zhou and colleagues describe a new database aiming to link an individual's genetic makeup to the phenotype they manifest. The authors have undoubtedly hit upon a critical question as the endlessly expanding glut of genomic sequence data has failed to result in dramatic improvements of patient outcomes, it is unclear if "My Personal Mutanome" has bridged this gap.

**Response:** We thank the Reviewer for the overall positive feedback regarding our study design and the constructive critiques. In the past two months weeks, we have

performed extensive additional analyses and new computational and experimental data to improve the utility of our “My Personal Mutanome” tools in multiple ways. The new data we now incorporated in the revised version of the manuscript are attached as below.

2. The first concern for any model or dataset lies in the fundamental input data upon which it is based. The authors integrated an expansive set of data encompassing ~500,000 somatic mutations, over 120,000 physical PPIs, and over 500,000 functional sites. The risk with such large datasets is that they are often (1) intrinsically biased due to sociological and funding prioritization, (2) of vastly differing quality depending on the methods and rigor applied to generate the data and (3) massively incomplete. It is unclear if the authors explored any of these potential issues or attempted to control for them.

**Response:** We thank the Reviewer for these critiques and we agree with some limitations. In our recent bioRxiv paper, we have demonstrated that PPI interface mutations provide novel insights into the functional consequences of disease variants/mutations and drug responses, and we validated them experimentally (Cheng et al., bioRxiv 2020). In our previous studies, we have demonstrated that somatic missense mutations are significantly enriched in protein functional sites (including ligand-protein binding sites, allosteric sites, and phosphorylation sites), which offers useful tools to prioritize likely functional mutations related to cancer and drug responses (Zhao et al., *Mol Cell Proteomics* 2016; Shen et al., *Am J Hu Genetics* 2017; Zhao et al., *Cancer Research* 2018). Several limitations, such as literature bias of PPIs, incompleteness and quality of data, have been extensively investigated and addressed in our previous studies (Zhao et al., *Mol Cell Proteomics* 2016; Shen et al., *Am J Hu Genetics* 2017; Zhao et al., *Cancer Research* 2018; Cheng et al., bioRxiv 2020). For

example, we found that somatic missense mutations are significantly enriched in computationally predicted PPI interfaces, consistent with experimental PPI interfaces from protein crystal structure data (please see the below responses to your Comment #3). To reply the Reviewer’s concern, we have intensified the rigor of computational analysis and provided new benchmarking validation data in the revised manuscript (page 5).

**References**

1. Cheng F, Zhao J, Wang Y, Lu W, Liu Z, Zhou Y, et al. Comprehensive characterization of protein-protein interaction network perturbations by human disease mutations. bioRxiv. doi: <https://doi.org/10.1101/2020.09.18.302588>
2. Zhao J, Cheng F, Wang Y, Arteaga CL, Zhao Z. Systematic prioritization of druggable mutations in ~5000 genomes across 16 cancer types using a structural genomics-based approach. Mol Cell Proteomics. 2016;15:642–56.
3. Shen Q, Cheng F, Song H, Lu W, Zhao J, An X, et al. Proteome-Scale Investigation of Protein Allosteric Regulation Perturbed by Somatic Mutations in 7,000 Cancer Genomes. Am J Hum Genet. 2017;100:5–20.
4. Zhao J, Cheng F, Zhao Z. Tissue-specific signaling networks rewired by major somatic mutations in human cancer revealed by proteome-wide discovery. Cancer Res. 2017;77:2810–21.

**3.** To identify interaction interfaces the authors used three very different sources of data, PDB, ECLAIR, and Interactome3D. While PDB contains experimentally derived structural data of the proteins, including in some cases the pair of interacting proteins in question, ECLAIR (which contributed the vast majority of the interface data) uses a machine learning approach to try to predict the interface between two proteins. Such approaches are fraught with problems in particular for protein lacking clear homologues or poorly studied proteins. It would be much more convincing to see how the model could perform with "gold standard" interface data from 3D models and then determining if predicted interface data such as that from ECLAIR could match the performance of the 3D models. What would be the impact of only using data from the resolved 3D models?

**Response:** We have compared the PPI interface mutation enrichment in three different databases separately in our recent bioRxiv paper. We found that somatic missense mutations are significantly enriched in PPI interfaces from both 3D models and ECLAIR (**Extended Figure 8 and 9**). As the resolved 3D models only contain approximately 10% human binary PPIs, we thus included ECLAIR data as well to increase coverage of

human proteome. As demonstrated in the original study (Meyer et al., *Nat Methods.* 2018), ECLAIR offer high-quality, computationally predicted PPI interfaces for experimentally validated PPIs. We have added more explanations in the revised manuscript.

**Extended Figure 8.** Distribution of somatic missense mutations in protein-protein interfaces versus non-interfaces across 33 cancer types from TCGA using all physical protein-protein interactions **with known crystal structures from the PDB database** ([www.rcsb.org](http://www.rcsb.org)). P-value < 2.2×10^-16^, two-sided Wilcoxon test) for all 33 cancer types.

**Extended Figure 9.** Distribution of somatic missense mutations in protein-protein interfaces versus non-interfaces across 33 cancer types from TCGA using physical protein-protein interactions with computationally predicted interfaces alone from the ECLAIR database. P-value < 2.2×10^-16^, two-sided Wilcoxon test) for all 33 cancer types.

**References**

1. Cheng F, Zhao J, Wang Y, Lu W, Liu Z, Zhou Y, et al. Comprehensive characterization of protein-protein interaction network perturbations by human disease mutations. bioRxiv. doi: <https://doi.org/10.1101/2020.09.18.302588>
2. Meyer MJ, Beltrán JF, Liang S, Fragoza R, Rumack A, Liang J, et al. Interactome INSIDER: a structural interactome browser for genomic studies. *Nat Methods*. 2018;15:107–14.
4. To evaluate the impact of a variant on a functional site the authors looked for mutations with 7 amino acids (upstream or downstream) of the functional site. It is unclear how they settled on this 15 amino-acid window or if they considered alternative window sizes. While clearly the amino acid sequence surrounding a site can impact the accessibility of a site, it is hard to believe that this is equal for all types of sites or all proteins. Did the authors attempt to verify the impact of any mutations flanking a functional site identified as potentially critical in their analysis? It would be relatively simple to demonstrate the impact of mutations surrounding a functional (e.g. phosphorylation) site using conventional biochemical approaches.

**Response:** We chose window size ±7 based on a previous study (Reimand et al., *Mol Syst Biol.* 2014). Because of the sparsity of somatic mutation data, the statistical analysis would be underpowered if we only consider mutations in the exact sites. To address this issue, we performed new analyses on phosphorylation sites and tested window sizes ±0 (exact site), ±1, ±3, ±5, and ±7. We computed the spearman correlation of the results for these window sizes. We found high correlations across window sizes ±3, ±5, and ±7. Therefore, we chose to keep the ±7 amino-acid window to cover more somatic mutations in the revised manuscript. We have added these new results in **Figure S5** and added more explanations in the revised manuscript (page 18).

**Reference**

1. Reimand J, Bader GD. Systematic analysis of somatic mutations in phosphorylation

signaling predicts novel cancer drivers. Mol Syst Biol. 2014;10:637.

5. Similarly, the authors look for an enrichment in mutations in the region identified to mediate protein-protein interactions. Did the authors test any of these interactions? Firstly, to demonstrate that they had correctly identified the interface region, and secondly that the mutations did indeed impact the interaction? Without such evidence it is hard to support the authors conclusion that these mutations are perturbing interactions and hence contributing to the observed phenotype.

**Response:** As we demonstrated in our recent bioRxiv paper (under minor revision by *Nature Genetics*), we experimentally validated the network effects of 13 PPI interface mutations (from 23 selected PPI interface mutations) using a well-established Y2H assay. As shown in **Extended Figure 10**, among 23 tested mutations, 17 (74%) led to lost PPIs or reduced the detected effects of PPIs, while 6 (26%) maintained the interactions predicted to be affected by the PPI interface mutations.

15

**Extended Figure 10.** The effect of mutations for 13 oncoPPIs were tested. Among the 23 tested mutations, 17 (74%) led to lost or reduced detection of PPIs (yellow and light blue).

We further experimentally validated that ALOX5 p.Met146Lys at the ALOX5-MAD1L1 interface and RXRA p.Ser427Phe at the RXRA-PPARG interface promoted significant tumor cell growth using cell line-based functional assays, providing a functional proof-of-concept of PPI interface mutations (**Extended Figure 11**).

**Extended Figure 11.** ALOX5 p.Met146Lys at the ALOX5-MAD1L1 interface and RXRA p.Ser427Phe at the RXRA-PPARG interface promote significant tumor cell growth using cell line-based functional assays.

**Reference**

**1.** Cheng F, Zhao J, Wang Y, Lu W, Liu Z, Zhou Y, et al. Comprehensive characterization of protein-protein interaction network perturbations by human disease mutations. bioRxiv. doi: <https://doi.org/10.1101/2020.09.18.302588>

**6.** For the drug responses, it is again unclear from the text how the authors associated a drug to a gene, or if they validated any of the connections they proposed. Can the authors provide either experimental data to show that the drug impact the gene they have mapped it to, or develop some sort of gold-standard reference set (not used in the training) and show how often they correctly paired drugs with target genes.

**Response:** Yes, we have used experimentally reported pharmacogenomics dataset to test drug responses correlated to PPI interface mutations and experimentally validated them using cell line-based functional assays (Cheng et al., bioRxiv 2020).

To test whether PPI interface mutations predict anticancer drug responses (**Extended Figure 12a**), we used ANOVA to determine if there is a significant difference between the cell lines of the PPI interface-mutated group and the PPI interface wild-type group in terms of their sensitivity/resistance (measured by the half-maximal inhibitory concentration [IC50]). We used experimentally reported drug pharmacogenomics profiles of over 1,000 cancer cell lines from the Genomics of Drug Sensitivity in Cancer (GDSC) database as the gold-standard reference set. We found that PPI interface-predicted mutations are highly correlated with sensitivity or resistance to multiple therapeutic agents. **Extended Figure 12b** shows that PPI interface mutations are highly correlated with the sensitivity or resistance of 66 clinically investigational or approved anticancer agents in cancer cell lines.

To assess better the clinical potential of the PPI-perturbing mutations, we further investigated their correlation with anticancer drug responses by analyzing the data from *in vivo* compound screens between ~1,000 patient-derived tumor xenograft (PDXs) models and 62 medications (including both monotherapy and combination therapy). In total, we found 2,808 significant correlations (P < 0.05, ANOVA test) between 49 medications and 1,411 putative PPI interface mutations (**Extended Figure 12c**).

Finally, we also validated that p.Ser427Phe-expressing Capan-2 was modestly susceptible to GSK0660 (IC50= 1.11 μM), when compared with empty vector (EV, IC50=8.41 μM) or WT (IC50= 2.51 μM)-transfected cells. A similar result was also obtained using the SW1990 cell line (EV: IC50 = 6.99 μM, WT: IC50=2.84 μM and p.Ser427Phe: IC50=1.80 μM, **Extended Figure 13a** and **13b**).

**Extended Figure 12.** (**a**) Experimental design of pharmacogenomics predicted by PPI-perturbing alleles. (**b**) Drug responses evaluated by mutation-perturbed PPIs (putative oncoPPIs) which harbor a statistically significant excess number of missense mutations at PPI interfaces by following a binomial distribution across 66 selected anticancer therapeutic agents in cancer cell lines. Each node denotes a specific oncoPPI. The size of a node denotes the p-value levels computed by ANOVA. Color of nodes represents three different types of PPIs (see Figure 1c legend). (**c**) Drug responses evaluated by oncoPPIs in the Patient-Derived Xenograft (PDX) models. (**d** & **e**) Highlighted examples of drug response for encorafenib and its combinations (LEE011 and encorafenib) predicted by interface mutations on VCL-FXR1 and BRAF-MAP2K1, respectively.

**Extended Figure 13.** Suppression of wild-type (WT) and mutant RXRA-driven cell proliferation by GSK0660, a potent PPARβ/δ antagonist. The details are provided in Cheng bioRxiv 2020 (Under minor revision by *Nature Genetics*).

**Reference**

1. Cheng F, Zhao J, Wang Y, Lu W, Liu Z, Zhou Y, et al. Comprehensive characterization of protein-protein interaction network perturbations by human disease mutations. bioRxiv. doi: <https://doi.org/10.1101/2020.09.18.302588>

**7.** In summary, while there is undoubtedly a compelling need for better tools to

functionalize genomic data, it is unclear if the authors have succeeded in meeting this goal. The methods used to select the data to incorporate in the analysis and how the authors showed that these datasets were the best to use are unclear. Better control using training and test sets to demonstrate the accuracy of their tool would go a long way to convincing the reader that this is a valuable and useful resource.

**Response:** We apologize that we didn’t add experimentally validation data in our original submission. In the past 2 months, we have performed extensive additional computational analyses and new experiments to meet the criticisms raised by the Reviewer. In particular, we intensified the rigor of our computational analysis and discussed more experimental data reported in our recent bioRxiv paper (Cheng et al., 2020) in the revised manuscript.

As a result of these extensive changes, we believe the manuscript has been considerably improved, and contains a set of highly unique observations that would be interest to the readership of *Genome Biology*. As stated by Editor in the end of the letter. “*This is our format for publishing database with major utility to a broad field of research, but that* ***do not provide significant biological insights***.” for **database article**.

**8.** Minor. HGNC reports 19,324 protein coding genes, yet the authors describe "21,759 proteins" (page 10 line 21). Could the authors clarify what they are considering as a unique protein in their study?

**Response:** The number 21,759 is the number of proteins, not unique protein coding gene number. We downloaded the gene information from HGNC (07/16/2019). We then downloaded the protein list from the UniProt database which contains gene to protein mappings. Some genes map to more than one protein as there are multiple protein isoforms for one gene. Therefore, the number of unique proteins is different from the number of unique genes. We have added the number of 19149 protein coding genes (the reminding 175 protein coding genes [19324-19149] don’t have any reported somatic mutations from TCGA database) in the revised manuscript.

**Second round of review**

**Reviewer 1**

The authors addressed my concerns.

**Reviewer 2**

The authors have made significant improvement to the manuscript particularly in the context of evaluation of features used to infer nodetic and edgetic effects. They have also included SIFT and PolyPhen scores which are useful predictors of pathogenic mutations. However, there are a few minor comments.

In response to comment #3, the authors mention "most CPTAC datasets for TCGA tumour types have been postponed for publication purposes. We thus only selected COAD (colon cancer) without publication restriction. We analyzed COAD phosphorus-proteomics data and did find relatively lower protein quantification in mutated phosphorylation sites than wild type sites (Figure S6, Extended Figure 4), suggesting loss of signaling by somatic missense mutations."

However, a large number of phospho-proteomics datasets from CPTAC Phase II (for example Breast and Ovarian Cancer) are currently published and not in any form of embargo, and have been used in many recent meta-analysis studies.

Nevertheless, the authors claim to see a difference in the sole dataset they analysed and "find relatively lower protein quantification in mutated phosphorylation sites than wild type sites (Figure S6, Extended Figure 4), suggesting loss of signaling by somatic missense mutations."

This result is a bit confusing as its unclear whether the authors are comparing overall protein abundance or phosphorylation levels at the mutated site. The expectation would be that mutation of the phosphorylation sites will disrupt the phosphorylation status of the protein.

Related to this analysis, the authors claim the following in the discussion: "We found that protein quantification is lowered in mutated phosphorylation sites than wild type sites (Figure S6) in COAD [60], showing proof-of-concept of functional impacts by phosphorylation site mutations." These are strong claims based on preliminary analysis of one dataset. The authors may wish to bring more clarity to this analysis in the manuscript and tone down the claims unless more analysis/data is provided.

In comment #7, the authors were unable to incorporate and evaluate FOLDx values in the context of their work and mention that "FOLDX is a protein structure- based webserver and we cannot get results from FOLDX for ~500K mutations in a short period due to high computational cost of FOLDX". Just to note, pre-calculated Foldx scores for all possible mutations in all human proteins with structure or reliable homology models are already available in Mutfunc database.

Finally, the current version of the MPM (My Personal Mutanome) database provides comprehensive annotations of mutations in tumours part of TCGA. It does not perform a personalised genome analysis as the title may suggest. Therefore, a more appropriate title should be considered.

**Reviewer 3:**

The authors have done a satisfactory job responding to the critiques.

**Authors Response**

**Point-by-point responses to the reviewers’ comments:**

**Responses to Review #2:**

The authors have made significant improvement to the manuscript particularly in the context of evaluation of features used to infer nodetic and edgetic effects. They have also included SIFT and PolyPhen scores which are useful predictors of pathogenic mutations. However, there are a few minor comments.

**1.** In response to comment #3, the authors mention "most CPTAC datasets for TCGA tumour types have been postponed for publication purposes. We thus only selected COAD (colon cancer) without publication restriction. We analyzed COAD phosphorus-proteomics data and did find relatively lower protein quantification in mutated phosphorylation sites than wild type sites (Figure S6, Extended Figure 4), suggesting loss of signaling by somatic missense mutations." However, a large number of phospho- proteomics datasets from CPTAC Phase II (for example Breast and Ovarian Cancer) are currently published and not in any form of embargo, and have been used in many recent meta-analysis studies.

**Response:** We have downloaded new CPTAC datasets and performed the same analysis for breast cancer and ovarian cancer. We found relatively lower protein quantification in mutated phosphorylation sites than wild type sites (**Figure S6**, **Extended Figure 1**) for both breast (BRCA, p=0.052) and ovarian cancer (OV, p=0.18); yet, the differences are slightly not significant compared to colon cancer (COAD, p=0.012). There are two possible explanations: (1) sample size of BRCA (n=54) and OV (n=10) is smaller than COAD (n=486); (2) mutation load of BRCA and OV is lower than COAD. We have added new findings and more explanations in the revised manuscript.

**Extended Figure 1.** Protein quantification is lowered in mutated phosphorylation sites than wild type sites in (**A**) breast invasive carcinoma (n=54), (**B**) colon adenocarcinoma (n=486), and (**C**) ovarian serous cystadenocarcinoma (n=10). We downloaded the site level phosphoproteome data from <http://www.linkedomics.org>. The data were divided into two groups: mutated phosphosites and wild type phosphosites. We then compared the expression levels of these two groups. The final distributions in the boxplots were derived from resampling.

**2.** Nevertheless, the authors claim to see a difference in the sole dataset they analysed and "find relatively lower protein quantification in mutated phosphorylation sites than

wild type sites (Figure S6, Extended Figure 4), suggesting loss of signaling by somatic missense mutations." This result is a bit confusing as its unclear whether the authors are comparing overall protein abundance or phosphorylation levels at the mutated site. The expectation would be that mutation of the phosphorylation sites will disrupt the phosphorylation status of the protein.

**Response:** We thank the reviewer for this critique. The analysis we performed was based on the phosphoproteomics data at the phosphosite level. We have made the claim clearer in the revised manuscript.

3. Related to this analysis, the authors claim the following in the discussion: "We found that protein quantification is lowered in mutated phosphorylation sites than wild type sites (Figure S6) in COAD [60], showing proof-of-concept of functional impacts by phosphorylation site mutations." These are strong claims based on preliminary analysis of one dataset. The authors may wish to bring more clarity to this analysis in the manuscript and tone down the claims unless more analysis/data is provided. **Response:** We thank the reviewer for this critique. We performed new additional analyses for breast cancer and ovarian cancer using available CPTAC datasets. We found that protein quantification is significantly lowered in mutated phosphorylation sites than wild type sites in COAD (p=0.012); yet, protein quantification is relatively lowered in mutated phosphorylation sites than wild type sites in breast (BRCA, p=0.052) and ovarian cancer (OV, p=0.18). We thus revised our claims: “*We found that protein quantification is significantly lowered in mutated phosphorylation sites than wild type sites in COAD (p=0.012); yet, protein quantification is relatively lowered in mutated phosphorylation sites than wild type sites in breast (BRCA, p=0.052) and ovarian cancer (OV, p=0.18). These observations reveal potential functional impacts of phosphorylation site mutations; further experimental validation is highly warranted using large-scale proteogenomics and phosphoproteomics datasets from cancer cell lines or tumor tissues*.” in page 19 of the revised manuscript.
4. In comment #7, the authors were unable to incorporate and evaluate FOLDx values in the context of their work and mention that "FOLDX is a protein structure- based

webserver and we cannot get results from FOLDX for ~500K mutations in a short period due to high computational cost of FOLDX". Just to note, pre-calculated Foldx scores for all possible mutations in all human proteins with structure or reliable homology models are already available in Mutfunc database.

**Response:** We downloaded the precalculated FoldX score (ddG) from the Mutfunc database (<http://www.mutfunc.com>) as suggested and added the scores to the database and cited the original publication (Wagih et al., *Mol Syst Biol* 2018). We also added the CADD score (Rentzsch et al., *Nucleic Acids Res* 2019) from https://cadd.gs.washington.edu/. In addition, we updated the UI to include visualizations for these scores (**Extended Figure 2**).

**Extended Figure 2.** Mutation scores, including SIFT, PolyPhen 2, CADD, and FoldX have been added to the mutation page.

**References**

Wagih O, Galardini M, Busby BP, Memon D, Typas A, Beltrao P. A resource of variant effect predictions of single nucleotide variants in model organisms. *Mol Syst Biol.* 2018;14:e8430.

Rentzsch P, Witten D, Cooper GM, Shendure J, Kircher M. CADD: Predicting the deleteriousness of variants throughout the human genome. *Nucleic Acids Res.* 2019;47:D886–94.

**5.** Finally, the current version of the MPM (My Personal Mutanome) database provides comprehensive annotations of mutations in tumours part of TCGA. It does not perform a personalised genome analysis as the title may suggest. Therefore, a more appropriate title should be considered.

**Response:** We thank the Reviewer for this critique. We didn’t highlight or perform a personalised genome analysis in the main text as the personalised genome analysis cannot provide the statistical validation for our proposed edgetic and nodetic hypotheses. In this study, we used the pooled tumor genome data (population data) to statistically illustrate potential implications of edgetic and nodetic models to infer possible driver events for tumorigenesis and anticancer drug responses. In fact, our MPM database can provide comprehensive annotation if the user uploads the mutation profiles from individual genome data. In addition to providing annotation for edgetic and nodetic impacts, we also implemented new FoldX and CADD scores for each mutation, which can be applied to mutation profiles from personal genomes. To respond the Reviewer’s concern, we have changed the original title as “*My personal mutanome: a computational genomic medicine platform for searching network perturbing alleles linking genotype to phenotype*”. In addition, we have added more explanations and discussion about the future work to enhance personalised genome analyses using our MPM database.
